# Efficacy of combination treatment using YHO-1701, an orally active STAT3 inhibitor, with molecular-targeted agents on cancer cell lines

**DOI:** 10.1038/s41598-021-86021-8

**Published:** 2021-03-23

**Authors:** Keisuke Taniguchi, Hiroaki Konishi, Akiko Yoshinaga, Momomi Tsugane, Hiroyuki Takahashi, Fukiko Nishisaka, Yoshiyuki Shishido, Akira Asai

**Affiliations:** 1 Yakult Central Institute, Yakult Honsha Co., Ltd., Tokyo, Japan; 2 Pharmaceutical Department, Yakult Honsha Co., Ltd., Tokyo, Japan; 3 Center for Drug Discovery, Graduate School of Pharmaceutical Sciences, University of Shizuoka, Shizuoka, Japan

**Keywords:** Targeted therapies, Pharmacology

## Abstract

Signal transducer and activator of transcription 3 (STAT3) plays a critical role in regulating cell growth, survival, and metastasis. STAT3 signaling is constitutively activated in various types of hematologic or solid malignancies. YHO-1701 has been developed as an orally available STAT3 inhibitor. Herein, YHO-1701 in combination with molecular-targeted agents was evaluated. Additive or synergistic effects were observed in a broad spectrum of “combination treatment + cell line” pairs. Of particular interest was the synergistic effect observed when YHO-1701 was combined with imatinib or dasatinib [breakpoint cluster region-abelson (BCR-ABL) inhibitors], osimertinib [epidermal growth factor receptor (EGFR) inhibitor], crizotinib, alectinib, or ceritinib [anaplastic lymphoma kinase (ALK) inhibitors]. The results further showed a close relationship between these synergistic effects and the cellular levels of the key molecules involved in the target pathways for YHO-1701 and each combination drug. The combination of YHO-1701 with alectinib resulted in significantly greater antitumor activity without exhibiting body weight loss in an NCI-H2228 [echinoderm microtubule-associated protein-like 4 (EML4)-ALK fusion] xenograft mouse model. Our results strongly suggest that the logical strategy in combination with the novel STAT3 inhibitor YHO-1701 and other mechanistically different targeted agents, could be a promising approach in future clinical settings.

## Introduction

Aberrant constitutive activation of signal transducer and activator of transcription 3 (STAT3) has been documented at a high frequency in various malignant tumors^1–5^. Persistent STAT3 activation has been attributed to the dysregulation of upstream tyrosine kinases and negative regulators in the STAT3 signaling pathway^6,7^. STAT3 phosphorylation is conducive to malignancy by upregulating the expression of pro-oncogenes, such as survivin, allowing tumor cells to survive and proliferate^1–5^. Overexpression of phosphorylated STAT3 occurs in numerous tumors^1–5^, suggesting that STAT3 inhibition is a promising approach for controlling cancers. STAT3 activation involves multiple signaling pathways within the tumor microenvironment, thus making the antiproliferative strategies of inhibitors targeting upstream molecules difficult. In other words, this implies that synergy by STAT3 inhibition may be expected with inhibitors to block these pathways.

**Figure 1:**
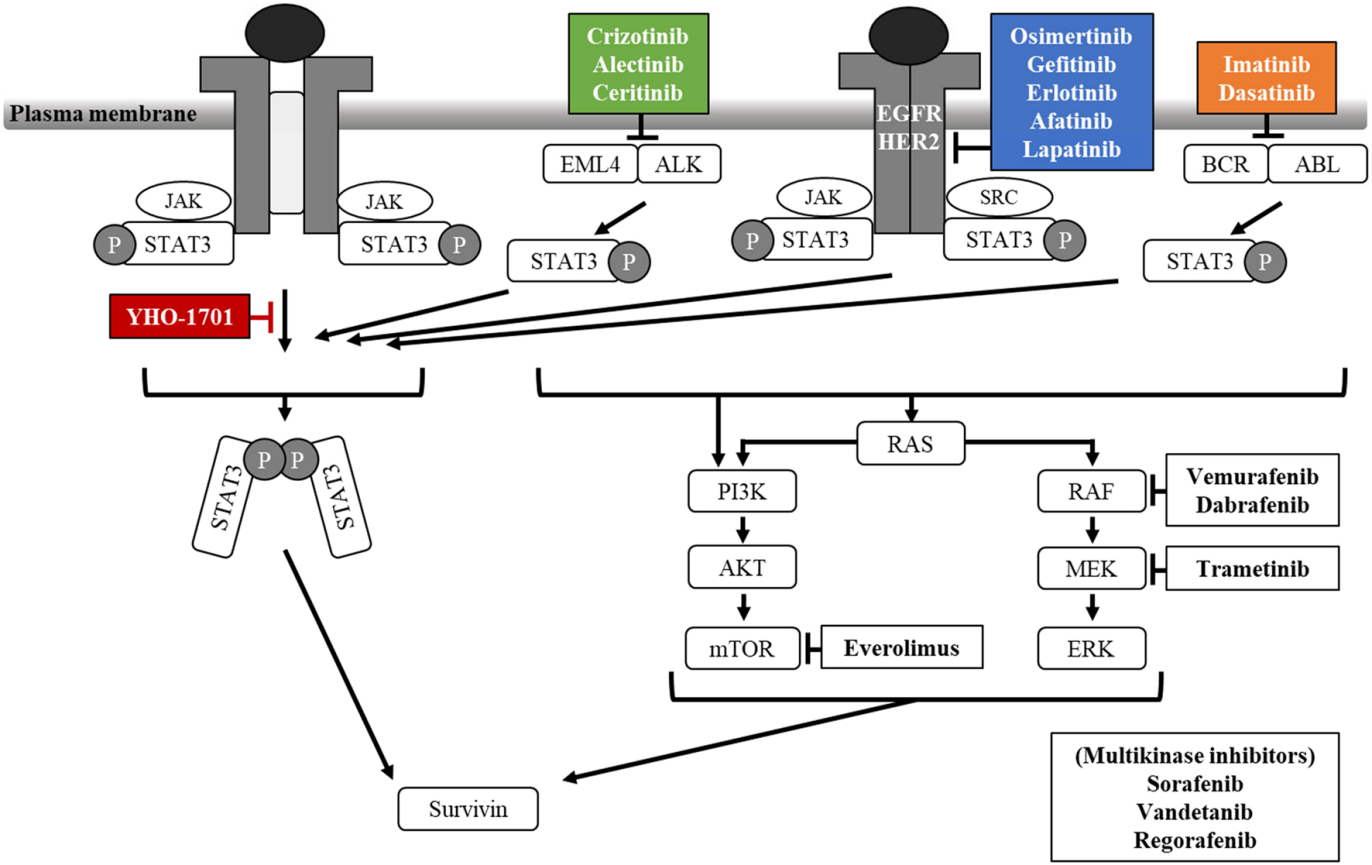
Schematic representation of the mechanism of growth inhibition by YHO-1701 and other molecular-targeted drugs. ER2, human epidermal growth factor receptor 2; JAK, Janus kinase; PI3K, phosphatidylinositol 3-kinase; AKT, protein kinase B; mTOR, mammalian target of rapamycin; MEK, mitogen-activated protein kinase kinase; ERK, extracellular signal-regulated kinase. Blunt arrows show molecular-targeted agents inhibiting relevant pathways.

We previously identified a novel orally active STAT3 inhibitor, YHO-1701. In SAS oral carcinoma cells, which are known for interleukin-6 signaling, YHO-1701 blocked multistep events accompanied by STAT3 dimerization, and also exhibited an enhanced antitumor effect with the multikinase inhibitor sorafenib^8^. These findings reveal that STAT3 is an attractive target, which motivated us to conduct further testing of a drug combination. STAT3 is a crucial convergence point in several ligand/receptor pathways and nonreceptor tyrosine kinase pathways; thus, the consequent cross-talk among these signaling pathways may be conducive to sensitivity to molecular-targeted drugs, such as breakpoint cluster region-abelson (BCR-ABL) inhibitors, epidermal growth factor receptor (EGFR) inhibitors, and anaplastic lymphoma kinase (ALK) inhibitors (Fig. 1). For example, although most patients with echinoderm microtubule-associated protein-like 4 (EML4)-ALK positive non-small cell lung cancer (NSCLC) derive advantages from treatment with ALK inhibitors, the clinical response to these drugs varies significantly among such individuals^9^. Cross-talking among the signaling pathways is complicated and requires further investigation; however, the underlying mechanisms of responses to ALK inhibitors have been identified only in recent years. These primarily include EML4-ALK gene aberrations (mutation or amplification)^10–15^. Another commonly observed mechanism is the upregulation of substitute tyrosine kinases, such as EGFR, KIT, and c-MET, that could bypass the ALK signaling pathway^14,16,17^. However, these findings do not necessarily explain all drug responses, and the molecular mechanisms underlying this insecurity remain uncertain. Therefore, further understanding of the signaling pathways is of immense importance to future clinical practice. We hypothesized that STAT3 signaling undertakes a vital role in limiting the antiproliferative response to various already-available targeted agents. Herein, we designed a combination strategy of molecular-targeted drugs with the new STAT3 inhibitor YHO-1701 to overcome the insufficiency in inducing potent antiproliferative responses. Furthermore, since STAT3 binds directly to the survivin promoter and regulates its expression^18^, we sought to determine the utility of the biomarker survivin for YHO-1701 treatment. Considering the aforementioned findings, targeting STAT3 signaling appears to be a novel approach to treat cancers.

## Results

### Identification of synergistic combinations in human cancer cell lines

**Figure 2:**
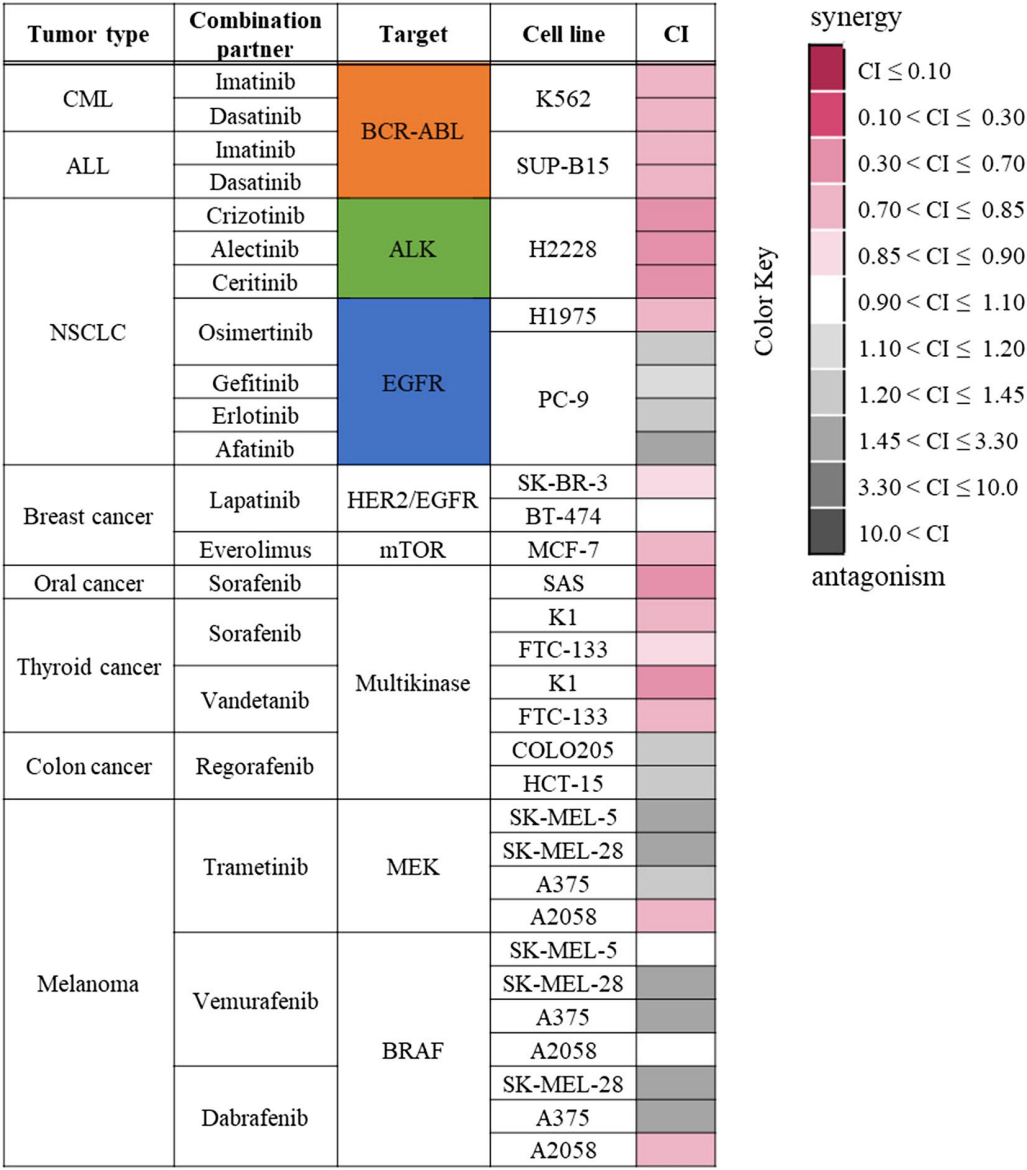
In vitro synergy of YHO-1701 in combination with already-available targeted agents for a total of 33 “combination + cell line” pairs. Drug interactions (synergism or antagonism) were analyzed using the combination index (CI) method. The antiproliferative effect of YHO-1701 and/or molecular-targeted agents was evaluated in a human cancer cell line panel using the WST-8 assay at 48 or 72 h of exposure. CI values were calculated and represented as a synergy “heat map” where a drug combination is synergistic (pink color) if CI ≤ 0.9; additive (white color) if CI is 0.9–1.1; and antagonistic (gray color) if CI > 1.1.

To expand our previous observations^8^, a total of 33 “combination + cell line” sets were evaluated with reference to previous research^19,20^. A heat map is shown in Fig. 2 for YHO-1701 in combination with the already-available targeted agents for hematologic or solid malignancies. The combination activity pattern was complex, and additive or synergistic effects were observed in approximately two-thirds of the total number of combination treatments given, whereas antagonism was observed in the other pairs. Interestingly, the YHO-1701/sorafenib combination exhibited an antiproliferative synergistic effect in SAS oral cancer cells as noted elsewhere^8^. This combination and combination with another multikinase inhibitor, vandetanib, also displayed synergistic inhibitory effects on the growth of both thyroid cancer cell lines. In contrast, YHO-1701 combined with trametinib (MEK inhibitor), vemurafenib, or dabrafenib (BRAF inhibitors) was not preferable, wherein only 4 of 11 drug–cell line pairs were additive or synergistic in the four melanoma cell lines. Combinations with BCR-ABL inhibitors against lymphoma cell lines and with ALK inhibitors against NSCLC cell lines were desirable, wherein all the drug–cell line pairs exhibited synergistic effects. Combinations with EGFR inhibitors exhibited different effects in the two NSCLC cell lines. Collectively, all combinations with osimertinib, gefitinib, erlotinib, or afatinib demonstrated antagonism in PC-9 cells harboring EGFR deletion (E746-A750), whereas the YHO-1701/osimertinib combination was synergistic in the NCI-H1975 (hereinafter “H1975”) cells harboring the EGFR mutation L858R plus T790M.

### Mechanistic analyses of synergistic effects in vitro

**Figure 3:**
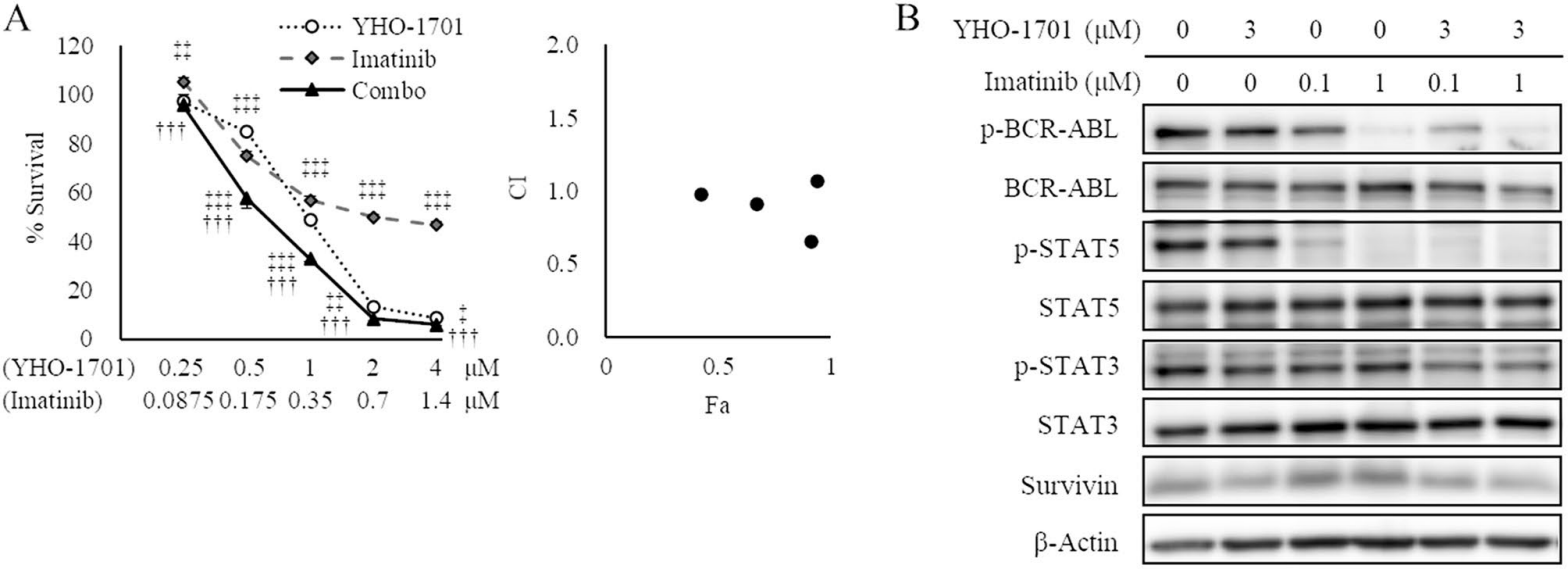
Synergism between YHO-1701 and imatinib in SUP-B15 cells. (**A**) The antiproliferative effect of YHO-1701 and/or imatinib was evaluated at 72 h of exposure. Dose–response curves are shown in the left panel. Combination index (CI) values are plotted as a function of fraction affected (Fa) in the right panel. Significance was determined with Tukey’s test. ^†††^p < 0.001 versus the imatinib group. ^‡^p < 0.05; ^‡‡^p < 0.01; and ^‡‡‡^p < 0.001 versus the YHO-1701 group. (**B**) Phosphorylation/activation patterns of crucial molecules on BCR-ABL and STAT3 signaling pathways were analyzed by western blotting. SUP-B15 cells were left untreated or treated with YHO-1701 and/or imatinib at the indicated doses for 24 h. The blots were probed with primary antibodies specific for the target proteins. Images were cropped for clarity, and full-length blots are presented in Supplementary Fig. S3.

**Figure 4:**
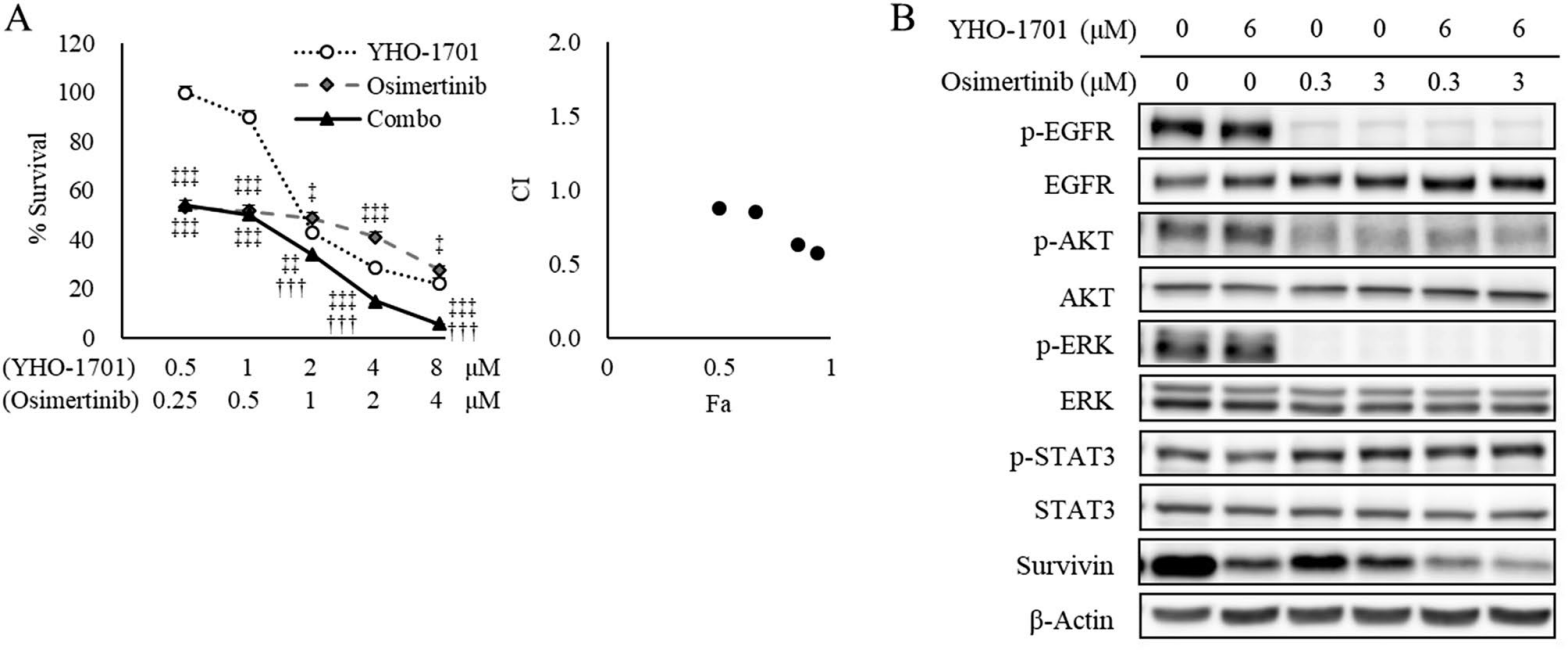
Synergistic antiproliferative interaction between YHO-1701 and osimertinib in H1975 cells. (**A**) The antiproliferative effect of YHO-1701 and/or osimertinib was examined at 72 h of exposure. Dose–response curves of YHO-1701 and/or osimertinib in H1975 cells (left panel) and their CI versus fraction affected (Fa) plots (right panel) are shown. Significance was determined with Tukey’s test. ^†††^p < 0.001 versus the osimertinib group. ^‡^p < 0.05; ^‡‡^p < 0.01; and ^‡‡‡^p < 0.001 versus the YHO-1701 group. (**B**) Phosphorylation/activation patterns of relevant molecules on EGFR and STAT3 signaling pathways were assessed by western blotting. H1975 cells were left untreated or treated with YHO-1701 and/or osimertinib at the indicated doses for 24 h. The blots were probed with the respective primary antibodies. Images were cropped for clarity, and full-length blots are displayed in Supplementary Fig. S3.

**Figure 5:**
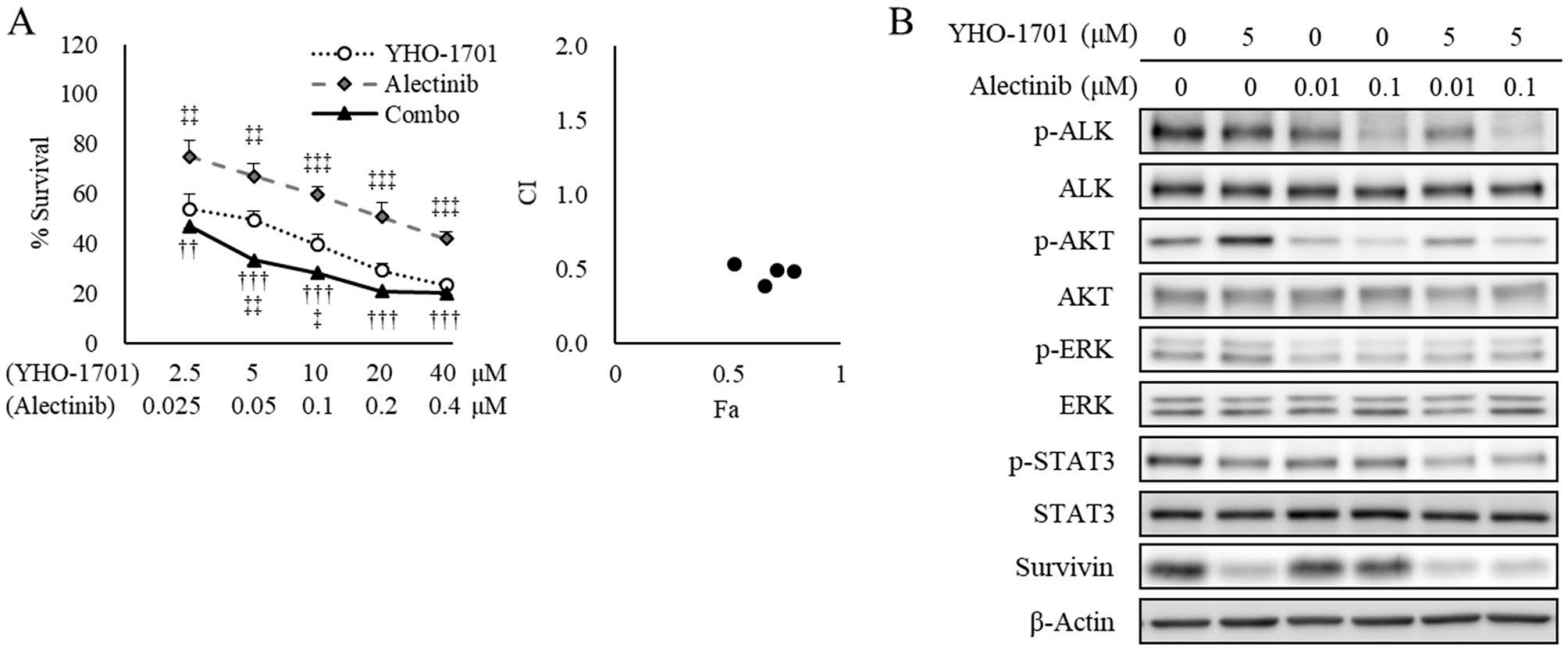
Synergism between YHO-1701 and alectinib in H2228 cells. (**A**) The antiproliferative effect of YHO-1701 and/or alectinib was investigated at 72 h of exposure. Dose–response curves are represented in the left panel. Combination index (CI) values are plotted as a function of fraction affected (Fa) in the right panel. Significance was determined with Tukey’s test. ^††^p < 0.01; ^†††^p < 0.001 versus the alectinib group. ^‡^p < 0.05; ^‡‡^p < 0.01; and ^‡‡‡^p < 0.001 versus the YHO-1701 group. (**B**) Phosphorylation/activation patterns of key molecules on ALK and STAT3 signaling pathways were examined by western blotting. H2228 cells were left untreated or treated with YHO-1701 and/or alectinib at the indicated doses for 24 h. The blots were probed with the respective primary antibodies. Cropped images are shown, and full-length blots are presented in Supplementary Fig. S3.

Of particular interest was the synergistic effect observed when YHO-1701 was combined with imatinib against SUP-B15 cells (BCR-ABL fusion), with osimertinib against H1975 cells (L858R/T790M EGFR double-mutant), and with alectinib against NCI-H2228 (hereinafter “H2228”) cells (EML4-ALK fusion) based on the median-effect method (Fig. 2) and its potential clinical use. Dose–response curves of YHO-1701 and/or imatinib in SUP-B15 cells, osimertinib in H1975, or alectinib in H2228 cells, and their respective combination index (CI) values are shown in Figs. 3A, 4A, and 5A. Additionally, the cell viability of all combinations is shown in Supplementary Table S1. To assess whether YHO-1701 and the combination partners are capable of inhibiting each target molecule at an earlier time point, cells were treated with single agents or their combination for 24 h. As expected, imatinib, osimertinib, and alectinib clearly suppressed their relevant molecules in each cell line (Figs. 3B, 4B, and 5B). Furthermore, YHO-1701 downregulated p-STAT3 expression in all cancer cell lines, consistent with the previous observation that YHO-1701 also inactivated the STAT3 signaling pathway in SAS cells^8^.

It has been reported that the third-generation EGFR inhibitor osimertinib, but not the first- or second-generation EGFR inhibitors gefitinib, erlotinib, or afatinib, is active against exon 19 and 21 mutations as well as the T790M mutation-positive disease^21–25^ and that the efficacy of EGFR inhibitors in EGFR-mutation NSCLC is limited by other salvage signals, including STAT3 and Src-YES-associated protein 1 (YAP1) signaling^26^. Reportedly, osimertinib failed to inhibit STAT3 signaling in EGFR^L858R/T790M^ mutant-expressing H1975 cells^26^. In accordance with this finding, in this study, osimertinib failed to suppress p-STAT3 in H1975 cells (Fig. 4B), and we demonstrated that combined treatment with osimertinib and YHO-1701 induced synergistic tumor growth inhibition (Figs. 2 and 4A). These findings corroborate the role of STAT3 signaling in providing a better therapeutic advantage against T790M mutation-positive NSCLC. However, we preliminary confirmed that the STAT3 activity is absent in PC-9 cells, as measured by western blot analysis, which is in agreement with the results of previous studies wherein PC-9 cells did not exhibit STAT3 activity^27,28^, providing the main reason for the different combination effects of osimertinib and YHO-1701 between PC-9 and H1975 cells (Fig. 2).

Although YHO-1701 induced p-AKT upregulation in H2228 cells, we did not observe any significant increase in the downstream target survivin level; conversely, it was lower than that in the vehicle-treated group (Fig. 5B). The survivin level was believed to be offset since YHO-1701 was also capable of suppressing survivin, consistent with our previous observation where it was abrogated through inhibiting STAT3 dimerization and/or STAT3 phosphorylation^8^. In contrast to moderate suppressions by single-drug treatments, the combination treatments strongly inhibited survivin levels in all sets (Figs. 3B, 4B, and 5B). After this experiment, we selected the “YHO-1701/alectinib + H2228” pair for the in vivo analysis based on particular research interests and its potential clinical use, in order to conduct further testing of the drug combination.

### YHO-1701 enhanced alectinib antitumor activity in an EML4-ALK fusion-positive H2228 xenograft model

**Figure 6:**
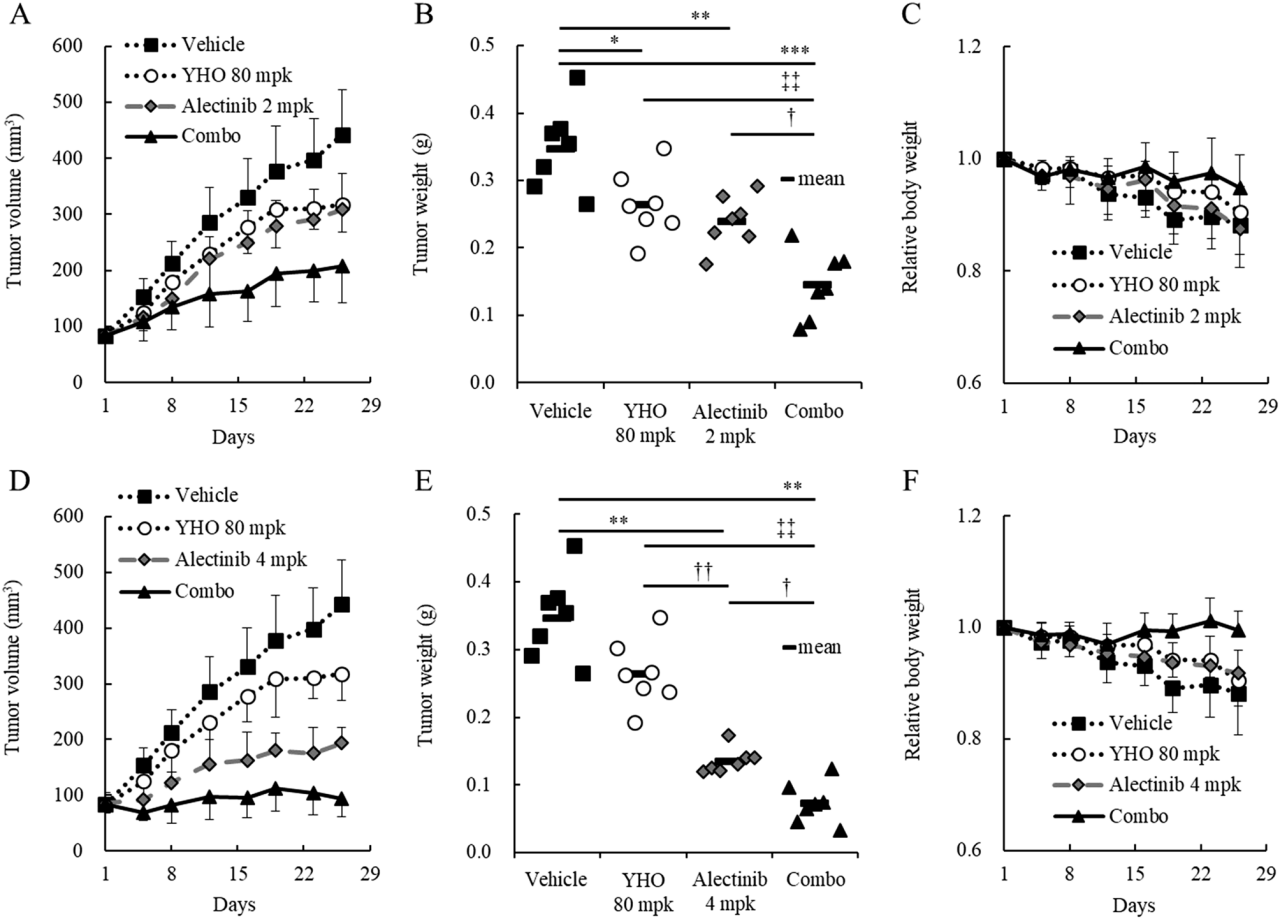
In vivo characterization of orally administered YHO-1701 when combined with alectinib at different levels of efficacy. Alectinib was used at 2 mg/kg/day (**A**–**C**) or 4 mg/kg/day (**D**–**F**). H2228 xenograft mice were randomized on day 1. Mice were treated with vehicle, YHO-1701, alectinib, or YHO-1701 + alectinib once daily for 4 weeks using a 5-day on, 2-day off schedule at indicated doses (n = 7). Changes in tumor volume (**A**,**D**), tumor weight on day 26 (**B**,**E**), and relative body weight (**C**,**F**). Significance was determined by Tukey’s test (**B**) or Steel–Dwass test (**E**). *p < 0.05; **p < 0.01; ***p < 0.001 versus the vehicle group. ^†^p < 0.05; ^††^p < 0.01 versus the alectinib group. ^‡‡^p < 0.01 versus the YHO-1701 group. mpk, milligrams per kilogram of body weight; YHO, YHO-1701.

We next evaluated whether YHO-1701 could enhance the antitumor effect of 2 mg/kg (Fig. 6A–C) or 4 mg/kg (Fig. 6D–F) of alectinib in H2228 xenograft mice. Our data revealed that 2 and 4 mg/kg of alectinib exhibited significant but different levels of antitumor responses, with IR values of 31.2% and 61.1%, respectively (p < 0.01). In other words, although 2 mg/kg of alectinib delayed tumor growth compared to that in the vehicle group; the tumors remained larger during the experimental period (Fig. 6A). In contrast, the average tumor volume remained almost stable in the 4 mg/kg group (Fig. 6D). Compared with the vehicle group on day 26, YHO-1701 alone exerted a moderate antitumor effect with an IR value of 24.0% (Fig. 6B,E). The plasma levels of YHO-1701 were higher than its in vitro IC_50_ value against H2228 cells for approximately 18 h and at least 24 h in mice that received single and 5-day repeated doses, respectively (Supplementary Fig. S1). YHO-1701 inhibited the in vitro proliferation of H2228 cells with an IC_50_ value of < 10 μM at 72 h, whereas the oral pharmacokinetic parameters in mice indicated that at the maximum plasma drug concentration (C_max_), the concentration of YHO-1701 was 17.7-fold higher with single dosing and 26.5-fold higher with repeated dosing than this IC_50_ value (Supplementary Fig. S1), suggesting adequate in vivo exposure to the compound. Adding YHO-1701 to alectinib resulted in superior tumor growth inhibitions to those observed in the respective monotherapy groups. The combination regimen led to 58.3% (Fig. 6B) and 79.2% (Fig. 6E) inhibition of H2228 tumor xenografts, which is suggestive of a potent combination efficacy, even under the conditions wherein alectinib monotherapy demonstrated broad-spectrum antitumor effects.

The body weight of H2228-bearing mice in the vehicle group decreased gradually, accompanied by tumor growth, similar to that observed in tumor-associated cachexia. Importantly, the H2228 xenograft-induced body weight loss was alleviated in the combination group because of tumor growth inhibition without exhibiting systemic toxicity (Fig. 6C,F). Moreover, no adverse effects in general conditions or any treatment-related macroscopic changes in major organs were induced by this therapeutically effective combination therapy, consistent with our previous observation^8^. These results indicate that this combination is effective for the treatment of EML4-ALK fusion-positive H2228 tumors and is not attributed to systemic toxicity.

**Figure 7:**
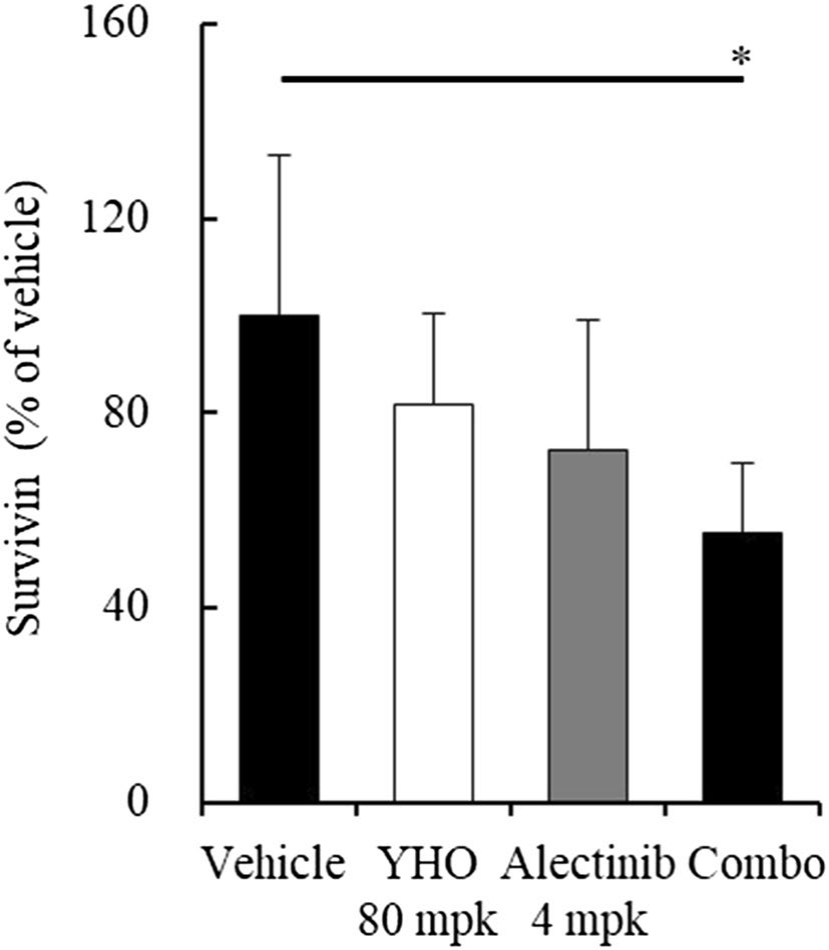
Downregulation of the downstream target survivin in tumor tissues. Lysates prepared from H2228 xenografts in Fig. 6E were subjected to further analyses. Survivin levels were determined by ELISA. Significance was determined by Tukey’s test. *p < 0.05 versus the vehicle group. mpk, milligrams per kilogram of body weight; YHO, YHO-1701.

Finally, we assessed the levels of survivin (the downstream target oncogene in the ALK and STAT3 pathways) in tumor tissues. In accordance with the in vitro analysis, alectinib 4 mg/kg and YHO-1701 monotherapies exhibited moderate suppression of survivin levels, despite the lack of significance. Moreover, the survivin suppression level was superior with the combination compared to that with either single agent (Fig. 7).

## Discussion

We previously discovered the STAT3 inhibitor YHO-1701, which exhibited a multistep regulation in the STAT3 signaling pathway by blocking the dimerization of STAT3 and demonstrated a strong antiproliferative effect and combination effect using the multikinase inhibitor sorafenib in cancer cell lines and in a human oral carcinoma SAS tumor xenograft model^8^. In response to this finding, we were interested in addressing whether the combination effect is applicable in subsequent situations with a diverse panel of molecular-targeted agents for hematologic or solid malignancies.

Here, we observed additive or synergistic effects in almost two-thirds of the total number of “combination + cell line” sets given. Considering its use in clinical practice, of particular interest was the synergistic effect observed when YHO-1701 was combined with imatinib, dasatinib (BCR-ABL inhibitors), osimertinib (EGFR inhibitor), crizotinib, alectinib, or ceritinib (ALK inhibitors). Compared with the moderate suppressions by single-drug treatments, the combination treatments strongly and reproducibly suppressed the major downstream molecule survivin in all sets (Figs. 3B, 4B, and 5B).

Based on our in vitro observations, we next explored the in vivo combination activity on the “YHO-1701/alectinib + H2228” set. Despite recent advances in research, the treatment regimen for EML4-ALK-positive patients with NSCLC remains unsatisfactory. For example, despite the fact that the NSCLC cell lines H3122 and H2228 harbor EML4-ALK, the antiproliferative efficacy of the ALK inhibitor TAE684 or crizotinib and ALK siRNA was insufficient against H2228 cells^29,30^. Furthermore, another research group emphasized the importance of dual interruption of the STAT3 and ERK signaling pathways in H2228 cells^9^, and we agree with their statement. In this study, we found that crizotinib and alectinib inhibited p-ERK, and the addition of the STAT3 inhibitor YHO-1701 to these agents resulted in greater downregulation of the STAT3-survivin signaling pathway and beneficial antiproliferative effects in H2228 cells (Figs. 2, 5, and S2). Meanwhile, crizotinib did not affect p-AKT (Fig. S2), consistent with the findings that neither TAE684 nor EML4-ALK depletion had a marked effect on p-AKT in H3122 or H2228 cells (“high” or “low” sensitive to ALK inhibition)^9^. This result implies that the AKT signal does not necessarily play a crucial role in the proliferation of these cancer cells. These findings support that although YHO-1701 induced the upregulation of p-AKT in H2228 cells, it resulted in clear growth inhibition. Moreover, sensitivity to ALK inhibitors is diminished by growth factors such as the hepatocyte growth factor^31^. In fact, another study showed that the MET signal salvaged the growth of H3122 and H2228 cells after treatment with alectinib, and its combination with a MET inhibitor, PHA-66752, potentiated the efficacy of alectinib^31^. Based on this finding, we anticipated that synergy would occur via STAT3 inhibition because STAT3 is known to be a more downstream point of convergence for various growth factors^32–35^. As expected, we confirmed that the addition of YHO-1701 to alectinib exhibited a promising combination effect against H2228 xenografts, where multiple factors in the tumor microenvironment can influence drug sensitivity. Additionally, this strategy may be applicable in combination with other ALK inhibitors (i.e., crizotinib and ceritinib) judging from their CI value that was comparable with that of the alectinib combination (Fig. 2).

To gain a better understanding of the contribution of YHO-1701 to enhance the antitumor properties of alectinib, YHO-1701 was combined with different doses of alectinib. The combination therapy of YHO-1701 with alectinib was found to be significantly effective against H2228 xenografts under both conditions, where the tumors remained larger and almost stable throughout the experimental period with alectinib monotherapy (Fig. 6A,D). This indicates that YHO-1701 offers an ideal and practical combination efficacy when used in combination with alectinib. Remarkably, in contrast with monotherapies, YHO-1701 plus alectinib diminished survivin levels in tumor tissues, suggesting that the superior antitumor activity of this combination is, at least partially, attributable to this downregulation. Regarding survivin, some studies have shown its potential characteristics as a useful biomarker^36,37^. We believe that a series of experimental results, including our previous data, could render survivin a promising marker in YHO-1701 treatment^8^. Our study has demonstrated additive or synergistic effects in two-thirds of the “combination + model” sets; however, the underlying mechanisms responsible for the combination effect remain to be completely elucidated. We still believe that the other combinations are also worth further investigation. In addition, we have highlighted the biological impact of YHO-1701 on cell proliferation and tumor growth. However, deregulation of the STAT3 signaling pathway not only facilitates cell proliferation, but also plays a key role in biological properties, including the invasion and metastasis of tumors^38,39^. Therefore, it is likely that STAT3 inhibition by YHO-1701 has pleiotropic effects in cancer therapy; tackling this subject is one of our future research directions.

Our study has a few limitations worth noting such as the use of cell line-based experiments. It is well known that cell lines are basically selected under typical two-dimensional cell culture conditions^40^. Nevertheless, patient-derived cancer cells (PDCs) used in three-dimensional spheroid cultures have advantages, as they often preserve the characteristics of the original tumor, including its heterogeneity and complexity^41,42^. As PDCs can also be applied to patient-derived xenograft models, further studies of YHO-1701 are of great interest. Another limitation is the small number of cell lines. Although we used 17 cell lines overall, the numbers for each type of cancer were relatively low because access to cell lines from patients with rare diseases was limited. That being said, to our knowledge, our study reports the first evidence of a successful combination therapy of ALK and STAT3 inhibitors against an EML4-ALK fusion-positive NSCLC in vivo model, which is potentially beneficial information for this patient population.

In conclusion, we clearly demonstrated that the antiproliferative activity of the combination of the novel STAT3 inhibitor YHO-1701 and a broad spectrum of mechanistically different targeted agents, including BCR-ABL, EGFR, and ALK inhibitors, was markedly greater than that of the respective agent alone. Although it is important to validate our findings using clinical specimens, our data suggest that the logical strategy of the combination of YHO-1701 with other mechanistically different targeted agents could be a promising approach for treating patients with leukemia and NSCLC in the future.

## Materials and methods

### Human cancer cell lines

The cell lines used in this study are listed in Supplementary Table S2; they were maintained according to the supplier’s instructions. Cultured cells were routinely tested for mycoplasma contamination using MycoAlert (Lonza, Walkersville, MD).

### Chemicals, antibodies, and reagents

YHO-1701 and gefitinib were synthesized at Yakult Honsha Co., Ltd. Other molecular-targeted agents were purchased based on their use in clinical practice and particular research interests for the combination experiments and are listed in Supplementary Table S3. In the in vitro assays, these agents were dissolved in dimethyl sulfoxide (DMSO). The final DMSO concentration was adjusted to 0.1%. In the in vivo study, YHO-1701 and alectinib were suspended in 0.5% methyl cellulose 400 cp solution and 0.5% HPMC/1 μmol/L hydrochloric acid (HCl) solution, respectively. Supplementary Table S4 lists the antibodies. HCl solution was purchased from FUJIFILM Wako Pure Chemical Corporation (Osaka, Japan). All other reagents were obtained from Sigma-Aldrich (St. Louis, MO), unless otherwise specified.

### Dose-response curves for IC_50_ determination

Dose-response curves were generated to determine the inhibitory concentration to achieve 50% cell death (IC_50_ values). Briefly, cells were grown overnight in 96-well plates and then left untreated or treated with test articles at various concentrations prepared by serial dilutions. After 48 or 72 h, the extent of cell viability was assessed by the WST-8 dye-based assay (Kishida chemical, Osaka, Japan) as described previously^43^. For each treatment condition, the mean values from triplicate wells were calculated and the dose–response curves plotted.

### Drug combination studies

The median-effect method was used to analyze the combination effects of YHO-1701 with molecular-targeted drugs comprising a diverse panel of standard-of-care agents^44^. Specifically, among the EGFR inhibitors tested, gefitinib, erlotinib, and afatinib are not clinically available for the treatment of patients with the T790M mutation in exon 20 of EGFR (H1975 cells). Osimertinib is the standard therapy for the treatment of patients with NSCLC with acquired EGFR T790M mutation, and its use is not limited to PC-9 cells harboring EGFR deletion (E746-A750). The same applies to other drug–cell line pairs; this is why the number of combinations is different among cell lines.

Briefly, test articles were combined in the same ratios in which both agents were prepared at a series of concentrations ranging from 4× , 2× , 1× , 0.5× , and 0.25× for IC_50_ values plus 0.1% DMSO alone. The nature of the interaction between both drugs was assessed under the condition that cells were treated simultaneously for 48 or 72 h. The antiproliferative potential of the combination over a concentration range was compared to that obtained for each agent alone, and a measure of the synergy, referred to as the CI, was calculated using a median-effect mathematical algorithm^41^. CalcuSyn (Biosoft, Ferguson, MO) was used to calculate the CI values. CI ≤ 0.9, 0.9–1.1, and > 1.1 represent synergism, additive effect, and antagonism, respectively. Triplicate wells were set up for each treatment condition.

### Western blotting

For analyzing the phosphorylation/activation pattern of relevant molecules, cancer cells were treated with YHO-1701 and/or imatinib, osimertinib, or alectinib for 24 h; following this, they were lysed using RIPA buffer containing protease and phosphatase inhibitors (Nacalai Tesque, Inc., Kyoto, Japan). Lysate concentrations were determined using the BCA protein assay kit (Pierce Chemical Co., Rockford, IL) and normalized for protein load. The lysate was then separated on SDS–polyacrylamide gel electrophoresis gels and transferred onto polyvinylidene fluoride membranes. The membrane was treated with primary antibodies overnight at 4 °C. Proteins were detected using horseradish peroxidase-conjugated secondary antibodies, visualized with an ECL kit (GE Healthcare Biosciences), and exposed to LAS-3000 (GE Healthcare Biosciences).

### Evaluation of antitumor activity in vivo

The antitumor efficacy of YHO-1701, in combination with alectinib was investigated in an H2228 xenograft model. Male BALB/c nude mice aged 6 weeks were purchased from Japan SLC, Inc. (Shizuoka, Japan). Tumor cells were inoculated subcutaneously into the right dorsal region of the mice. When the mean tumor volume reached approximately 50–100 mm^3^ (day 1), they were randomly allocated to the following groups (n = 7): a vehicle group; a YHO-1701 monotherapy group (80 mg/kg); an alectinib monotherapy group (2 or 4 mg/kg alectinib); and a combination group. Treatment was started on day 1; the test compounds were administered orally using a 5-day-on/2-day-off × 4 cycle schedule. Tumor growth was monitored until day 26 by measuring two perpendicular diameters using a digital caliper (Mitutoyo, Kanagawa, Japan), and tumor volume was calculated as described earlier^45,46^. On day 26, xenograft tumors were weighed and snap-frozen 24 h after the last dose for the proof-of-concept study (see below). The antitumor efficacy was expressed based on tumor weight at day 26 as the percentage tumor growth inhibition (% IR), which was calculated using the formula: IR (%) = (1 − mean weight of the treated tumor/mean tumor weight in the vehicle group) × 100. Body weight was monitored twice a week to assess the tolerability of the combination therapy. The relative body weight (RBW) at day n was calculated using the following formula: RBW = body weight on day n/body weight on day 1.

The plasma YHO‐1701 concentrations were determined with LC‐MS/MS, as described previously^8^. Briefly, non–tumor‐bearing mice were orally administered either single (n = 3) or 5-day repeated (n = 6) doses of YHO‐1701 at 80 mg/kg, and C_max_ values were calculated using the mean values.

### Dose level of alectinib in vivo

Here, we selected relatively low doses of alectinib to identify the optimal combination that might otherwise have been visualized by more predominant antitumor responses at higher dose levels. Therefore, we did not focus on comparative activities and toxicity of monotherapy in this study.

### Proof-of-concept in tumor xenografts

The enzyme-linked immunosorbent assay (ELISA) of survivin, Pathscan Total Survivin Sandwich ELISA (Cell Signaling Technology, Beverly, MA) was used according to the manufacturer’s instructions. Briefly, tumor lysates were prepared in the same buffer that was used for western blotting. Lysates were adjusted to a concentration of 10 mg/mL protein, and an equal amount (100 μL/well) of total protein from each sample was incubated overnight at 4 °C. After washing the plate, the detection antibody (100 μL/well) was added and incubated at 37 °C for 1 h. Binding was detected using a horseradish peroxidase–linked streptavidin antibody and TMB substrate solution. The reaction product was measured at 450 nm (SpectraMax Plus 384, Molecular Devices Corp., Sunnyvale, CA). Survivin levels were quantified and represented as the percentage of the vehicle group.

### Statistical analysis

Data are presented as mean ± standard deviation (SD). Statistical analyses were performed using the SAS software (version 8.2, SAS Institute Japan Ltd., Tokyo, Japan) for tumor weight or the JMP software (version 14.3.0, SAS Institute Japan Ltd.) for in vitro antiproliferative effects and survivin levels in tumor tissues. Either a Tukey’s test or a Steel–Dwass test (Bartlett’s test; p < 0.05) was used for detecting statistical differences between individual groups. P values < 0.05 were considered statistically significant.

### Ethics statement

All animal studies were conducted in an Association for Assessment and Accreditation of Laboratory Animal Care (AAALAC)-accredited facility in accordance with the Guidelines of the Yakult Central Institute and protocols approved by the Animal Experimental Committee of the Yakult Central Institute (#18-0021; approved May 31, 2018). All animal work was performed in accordance with Animal Research: Reporting of In Vivo Experiments (ARRIVE) guidelines and regulations.

## Supplementary Information

Supplementary Information.
