# Supplementary material for: Efficacy of combination treatment using YHO-1701, an orally active STAT3 inhibitor, with molecular-targeted agents on cancer cell lines
Source: Sci Rep. 2021 Mar 23;11:6685. doi: 10.1038/s41598-021-86021-8 (PMC7988006; doi:10.1038/s41598-021-86021-8)

Title page

Title:

Efficacy of combination treatment using YHO-1701, an orally active STAT3 inhibitor, with molecular-targeted agents on cancer cell lines

Authors' names:

Keisuke Taniguchi[1], Hiroaki Konishi[1], Akiko Yoshinaga[1], Momomi Tsugane[1], Hiroyuki Takahashi[2], Fukiko Nishisaka[1, 3], Yoshiyuki Shishido[1], Akira Asai[3]

Authors' affiliations:

1 Yakult Central Institute, Yakult Honsha Co., Ltd., Tokyo; 2 Pharmaceutical Department, Yakult Honsha Co., Ltd., Tokyo; 3 Center for Drug Discovery, Graduate School of Pharmaceutical Sciences, University of Shizuoka, Shizuoka, Japan.

Corresponding author:

Keisuke Taniguchi, Pharmaceutical Research Department, Yakult Central Institute, Yakult Honsha Co., Ltd., 5-11 Izumi, Kunitachi-shi, Tokyo 186-8650, Japan. Phone: +81425778960. Fax: +81425773020. Email: keisuke-taniguchi@yakult.co.jp

Supplementary Information

Supplementary Table S1. Cell viability of different cell lines treated with YHO-1701 and/or already-available targeted agents at indicated doses.

The antiproliferative effects of YHO-1701 and/or molecular-targeted agents were evaluated in a human cancer cell line panel using the WST-8 assay at 48 or 72 h of exposure. Representative data are shown of at least three independent experiments.

**K562** YHO-1701 dose (μM)	Imatinib dose (μM)	% Survival (YHO-1701)	% Survival (Imatinib)	% Survival (Combo)
0.5	0.075	90	88	76
1	0.15	53	63	35
2	0.3	41	34	17
4	0.6	37	19	10
8	1.2	25	15	7

**K562** YHO-1701 dose (μM)	Dasatinib dose (nM)	% Survival (YHO-1701)	% Survival (Dasatinib)	% Survival (Combo)
0.375	0.075	92	84	81
0.75	0.15	67	69	51
1.5	0.3	38	42	18
3	0.6	31	20	8
6	1.2	25	14	9

**SUP-B15** YHO-1701 dose (μM)	Imatinib dose (μM)	% Survival (YHO-1701)	% Survival (Imatinib)	% Survival (Combo)
0.25	0.0875	97	105	96
0.5	0.175	85	75	57
1	0.35	49	57	33
2	0.7	13	50	8
4	1.4	8	47	6

**SUP-B15**<br>YHO-1701 dose<br>(µM)	Dasatinib dose<br>(nM)	% Survival<br>(YHO-1701)	% Survival<br>(Dasatinib)	% Survival<br>(Combo)
0.25	7.5	94	51	45
0.5	15	84	50	41
1	30	49	45	28
2	60	13	30	10
4	120	9	23	6

**H2228**<br>YHO-1701 dose<br>(µM)	Crizotinib dose<br>(µM)	% Survival<br>(YHO-1701)	% Survival<br>(Crizotinib)	% Survival<br>(Combo)
2.5	0.25	70	68	48
5	0.5	63	66	33
10	1	48	56	25
20	2	43	43	21
40	4	33	19	6

**H2228**<br>YHO-1701 dose<br>(µM)	Alectinib dose<br>(µM)	% Survival<br>(YHO-1701)	% Survival<br>(Alectinib)	% Survival<br>(Combo)
2.5	0.025	54	75	47
5	0.05	49	67	33
10	0.1	39	60	28
20	0.2	29	51	21
40	0.4	23	42	20

**H2228**<br>YHO-1701 dose<br>(µM)	Ceritinib dose<br>(µM)	% Survival<br>(YHO-1701)	% Survival<br>(Ceritinib)	% Survival<br>(Combo)
2.5	0.125	62	75	46
5	0.25	60	73	33
10	0.5	46	65	27
20	1	40	59	21
40	2	30	41	17

**H1975** YHO-1701 dose (μM)	Osimertinib dose (μM)	% Survival (YHO-1701)	% Survival (Osimertinib)	% Survival (Combo)
0.5	0.25	100	53	54
1	0.5	90	52	50
2	1	43	49	34
4	2	28	41	15
8	4	22	28	6

**PC-9** YHO-1701 dose (μM)	Osimertinib dose (nM)	% Survival (YHO-1701)	% Survival (Osimertinib)	% Survival (Combo)
0.25	1.25	94	91	95
0.5	2.5	87	82	70
1	5	42	64	28
2	10	23	42	18
4	20	12	28	12

**PC-9** YHO-1701 dose (μM)	Gefitinib dose (nM)	% Survival (YHO-1701)	% Survival (Gefitinib)	% Survival (Combo)
0.25	2.5	88	88	101
0.5	5	78	81	78
1	10	42	73	39
2	20	23	58	20
4	40	10	40	12

**PC-9** YHO-1701 dose (μM)	Erlotinib dose (nM)	% Survival (YHO-1701)	% Survival (Erlotinib)	% Survival (Combo)
0.25	5	95	87	90
0.5	10	81	76	69
1	20	40	57	33
2	40	23	39	17
4	80	10	28	12

**PC-9**<br>YHO-1701 dose (µM)	Afatinib dose (nM)	% Survival (YHO-1701)	% Survival (Afatinib)	% Survival (Combo)
0.25	0.25	94	90	102
0.5	0.5	85	86	81
1	1	43	63	33
2	2	24	35	20
4	4	12	28	12

**SK-BR-3**<br>YHO-1701 dose (µM)	Lapatinib dose (nM)	% Survival (YHO-1701)	% Survival (Lapatinib)	% Survival (Combo)
0.25	12.5	75	76	67
0.5	25	63	67	53
1	50	51	57	41
2	100	36	49	28
4	200	25	42	19

**BT-474**<br>YHO-1701 dose (µM)	Lapatinib dose (nM)	% Survival (YHO-1701)	% Survival (Lapatinib)	% Survival (Combo)
0.25	5	82	99	88
0.5	10	78	69	60
1	20	55	46	32
2	40	35	32	22
4	80	33	22	21

**MCF-7**<br>YHO-1701 dose (µM)	Everolimus dose (nM)	% Survival (YHO-1701)	% Survival (Everolimus)	% Survival (Combo)
0.5	0.375	94	71	67
1	0.75	80	59	51
2	1.5	47	52	36
4	3	31	47	23
8	6	24	45	20

**SAS**<br>YHO-1701 dose<br>(μM)	Sorafenib dose<br>(μM)	% Survival<br>(YHO-1701)	% Survival<br>(Sorafenib)	% Survival<br>(Combo)
0.125	0.75	103	82	75
0.25	1.5	95	70	37
0.5	3	61	47	13
1	6	39	19	14
2	12	15	15	12

**K1**<br>YHO-1701 dose<br>(μM)	Sorafenib dose<br>(μM)	% Survival<br>(YHO-1701)	% Survival<br>(Sorafenib)	% Survival<br>(Combo)
1.75	2.5	80	77	80
3.5	5	79	73	44
7	10	46	39	10
14	20	41	8	1
28	40	23	0	3

**FTC-133**<br>YHO-1701 dose<br>(μM)	Sorafenib dose<br>(μM)	% Survival<br>(YHO-1701)	% Survival<br>(Sorafenib)	% Survival<br>(Combo)
0.625	2	77	83	64
1.25	4	51	72	43
2.5	8	43	43	27
5	16	43	19	12
10	32	29	1	1

**K1**<br>YHO-1701 dose<br>(μM)	Vandetanib dose<br>(μM)	% Survival<br>(YHO-1701)	% Survival<br>(Vandetanib)	% Survival<br>(Combo)
1.75	3.75	77	110	79
3.5	7.5	75	111	29
7	15	45	42	3
14	30	39	1	1
28	60	22	0	0

**FTC-133**<br>YHO-1701 dose (µM)	Vandetanib dose (µM)	% Survival (YHO-1701)	% Survival (Vandetanib)	% Survival (Combo)
0.625	3.75	83	96	77
1.25	7.5	55	91	41
2.5	15	47	44	27
5	30	45	9	5
10	60	32	0	0

**COLO205**<br>YHO-1701 dose (µM)	Regorafenib dose (µM)	% Survival (YHO-1701)	% Survival (Regorafenib)	% Survival (Combo)
0.25	0.375	89	92	84
0.5	0.75	69	86	64
1	1.5	47	72	38
2	3	23	53	20
4	6	16	27	16

**HCT-15**<br>YHO-1701 dose (µM)	Regorafenib dose (µM)	% Survival (YHO-1701)	% Survival (Regorafenib)	% Survival (Combo)
0.25	1.25	95	89	79
0.5	2.5	81	72	57
1	5	58	48	35
2	10	25	29	16
4	20	18	11	12

**SK-MEL-5**<br>YHO-1701 dose (µM)	Trametinib dose (nM)	% Survival (YHO-1701)	% Survival (Trametinib)	% Survival (Combo)
0.625	0.5	87	61	54
1.25	1	52	46	34
2.5	2	48	40	24
5	4	35	33	22
10	8	13	33	20

**SK-MEL-28**<br>YHO-1701 dose<br>(µM)	Trametinib dose<br>(nM)	% Survival<br>(YHO-1701)	% Survival<br>(Trametinib)	% Survival<br>(Combo)
2.5	0.5	74	98	72
5	1	65	71	63
10	2	42	47	59
20	4	28	35	37
40	8	21	26	27

**A375**<br>YHO-1701 dose<br>(µM)	Trametinib dose<br>(nM)	% Survival<br>(YHO-1701)	% Survival<br>(Trametinib)	% Survival<br>(Combo)
0.125	0.125	104	100	101
0.25	0.25	101	96	92
0.5	0.5	68	78	57
1	1	43	33	34
2	2	28	22	17

**A2058**<br>YHO-1701 dose<br>(µM)	Trametinib dose<br>(nM)	% Survival<br>(YHO-1701)	% Survival<br>(Trametinib)	% Survival<br>(Combo)
0.175	1.25	99	91	90
0.35	2.5	96	81	75
0.7	5	62	68	52
1.4	10	36	53	30
2.8	20	33	48	25

**SK-MEL-5**<br>YHO-1701 dose<br>(µM)	Vemurafenib dose<br>(µM)	% Survival<br>(YHO-1701)	% Survival<br>(Vemurafenib)	% Survival<br>(Combo)
0.625	0.25	84	56	52
1.25	0.5	53	45	36
2.5	1	50	40	30
5	2	38	36	29
10	4	20	30	22

**SK-MEL-28**<br>YHO-1701 dose<br>(μM)	Vemurafenib dose<br>(μM)	% Survival<br>(YHO-1701)	% Survival<br>(Vemurafenib)	% Survival<br>(Combo)
2.5	0.075	75	89	65
5	0.15	66	70	57
10	0.3	42	51	53
20	0.6	28	38	33
40	1.2	22	31	29

**A375**<br>YHO-1701 dose<br>(μM)	Vemurafenib dose<br>(μM)	% Survival<br>(YHO-1701)	% Survival<br>(Vemurafenib)	% Survival<br>(Combo)
0.125	0.0125	98	100	100
0.25	0.025	93	82	84
0.5	0.05	63	59	56
1	0.1	42	35	51
2	0.2	30	26	23

**A2058**<br>YHO-1701 dose<br>(μM)	Vemurafenib dose<br>(μM)	% Survival<br>(YHO-1701)	% Survival<br>(Vemurafenib)	% Survival<br>(Combo)
0.175	1.25	102	70	71
0.35	2.5	96	59	53
0.7	5	59	47	35
1.4	10	36	37	20
2.8	20	32	19	13

**SK-MEL-28**<br>YHO-1701 dose<br>(μM)	Dabrafenib dose<br>(nM)	% Survival<br>(YHO-1701)	% Survival<br>(Dabrafenib)	% Survival<br>(Combo)
2.5	2.5	73	81	64
5	5	65	62	58
10	10	39	47	54
20	20	27	36	37
40	40	21	32	29

**A375**<br>YHO-1701 dose<br>(μM)	Dabrafenib dose<br>(nM)	% Survival<br>(YHO-1701)	% Survival<br>(Dabrafenib)	% Survival<br>(Combo)
0.125	1.25	101	76	78
0.25	2.5	90	56	53
0.5	5	56	37	36
1	10	36	25	32
2	20	29	21	17

**A2058**<br>YHO-1701 dose<br>(μM)	Dabrafenib dose<br>(μM)	% Survival<br>(YHO-1701)	% Survival<br>(Dabrafenib)	% Survival<br>(Combo)
0.7	12.5	63	67	55
1.4	25	37	61	40
2.8	50	33	47	18
5.6	100	23	23	5

Supplementary Table S2. List of cancer cell lines and their origins used in this study.

Cancer type	Cell line	Supplier	Culture medium
BCR-ABL-positive leukemia	K562	ATCC	IMDM/10% FBS
	SUP-B15	ATCC	IMDM/20% FBS + 0.05mM 2-Mercaptoethanol
EML4-ALK-positive NSCLC	NCI-H2228 (H2228)	ATCC	RPMI1640/10% FBS
EGFR mutation-positive NSCLC	NCI-H1975 (H1975)	ATCC	RPMI1640/10% FBS
	PC-9	ECACC	RPMI1640/10% FBS
Breast	SK-BR-3	ATCC	RPMI1640/10% FBS
	BT-474	ATCC	RPMI1640/10% FBS
	MCF-7	ATCC	DMEM/10% FBS
Oral	SAS	JCRB	DMEM/F12 /10% FBS
Thyroid	K1	ECACC	IMDM/10% FBS
	FTC-133	ECACC	IMDM/10% FBS
Colon	COLO205	ATCC	RPMI 1640/10% FBS
	HCT-15	ATCC	RPMI1640/10% FBS
Melanoma	SK-MEL-5	ATCC	DMEM/10% FBS
	SK-MEL-28	ATCC	DMEM/10% FBS
	A375	ATCC	DMEM/10% FBS
	A2058	ATCC	DMEM/10% FBS

ATCC, American Type Culture Collection (Manassas, VA); ECACC, European Collection of Authenticated Cell Cultures (Salisbury, UK); JCRB, Japanese Collection of Research Bioresources (Osaka, Japan).

Supplementary Table S3. List of already-available targeted agents used in this study.

Molecular-targeted agent	Drug target	Company and catalog number
Imatinib	BCR-ABL	Cell Signaling Technologies (No. 9084)
Dasatinib	BCR-ABL	Bio Vision (No. 1586)
Crizotinib	ALK	LC Laboratories (No. C-7900)
Alectinib	ALK	Selleck Chemicals (No. S2762)
Ceritinib	ALK	Active Biochem (No. A-1189)
Osimertinib	EGFR	Selleck Chemicals (No. S7297)
Gefitinib *	EGFR	Yakult Honsha Co., Ltd.
Everolimus	mTOR	LC Laboratories (No. E-4040)
Erlotinib	EGFR	Santa Cruz Biotechnologies (No. sc-202154)
Afatinib	EGFR	Santa Cruz Biotechnologies (No. sc-364398)
Lapatinib	HER2/EGFR	Santa Cruz Biotechnologies (No. sc-202205)
Sorafenib	Multi Kinases	Cayman Chemical (No. 10009644)
Vandetanib	Multi Kinases	Santa Cruz Biotechnologies (No. sc-220364)
Regorafenib	Multi Kinases	Active Biochem (No. A-1486)
Trametinib	MEK	LC Laboratories (No. T-8123)
Vemurafenib	BRAF	LC Laboratories (No. V-2800)
Dabrafenib	BRAF	Santa Cruz Biotechnologies (No. sc-364477)

*: Gefitinib was synthesized at Yakult Honsha Co., Ltd.

Supplementary Table S4. List of primary antibodies, including company catalog number.

Western blotting primary antibody	Dilution	Company and catalog number
Rabbit anti-p-ALK (Y1278/Y1282/Y1283)	1:1000	Cell Signaling Technologies (No. 3983)
Rabbit anti-ALK	1:1000	Cell Signaling Technologies (No. 3633)
Rabbit anti-p-AKT (S473)	1:3000	Cell Signaling Technologies (No. 4058)
Rabbit anti-p-ERK1/2 (T202/Y204)	1:1000	Cell Signaling Technologies (No. 4370)
Rabbit anti-ERK1/2	1:1000	Cell Signaling Technologies (No. 9102)
Rabbit anti-p-STAT3 (Y705)	1:1000	Cell Signaling Technologies (No. 9131)
Rabbit anti-STAT3	1:2000	Cell Signaling Technologies (No. 4904)
Rabbit anti-p-c-Abl (Y245)	1:1000	Cell Signaling Technologies (No. 2861)
Rabbit anti-c-Abl	1:1000	Cell Signaling Technologies (No. 2862)
Rabbit anti-p-EGFR (Y1068)	1:1000	Cell Signaling Technologies (No. 3777)
Rabbit anti-EGFR	1:1000	Cell Signaling Technologies (No. 4267)
Rabbit anti-p-STAT5 (Y694)	1:1000	Cell Signaling Technologies (No. 9351)
Rabbit anti-AKT	1:1000	Santa Cruz Biotechnologies (No. sc-8312)
Rabbit anti-STAT5	1:1000	Santa Cruz Biotechnologies (No. sc-835)
Rabbit anti-survivin	1:1000	R&D Systems (No. AF886)
Mouse anti-β-actin	1:5000	Sigma-Aldrich (No. A5316)

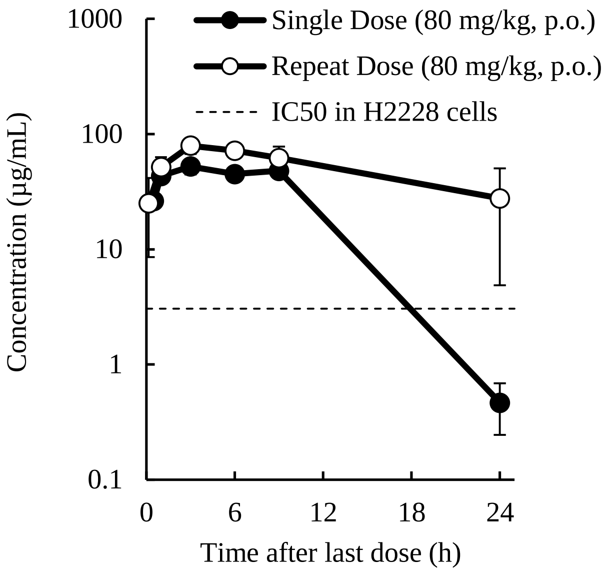

Supplementary Figure S1. YHO-1701 concentration-time profile in plasma after oral administration of single and repeat doses in mice.

The plasma concentration-time course of YHO-1701 (80 mg/kg) was shown in mice following single (n = 3) and 5-day repeated (n = 6) oral administrations. Blood samples were collected at 6 intervals. Data represent mean ± SD. The antiproliferative effect of YHO-1701 was evaluated in H2228 cells using the WST-8 assay after 72 h of exposure. Experiments were performed in triplicates. The horizontal dotted line at 3.05 µg/mL (6.55 µM) represents the *in vitro* IC_{50} for H2228 cells.

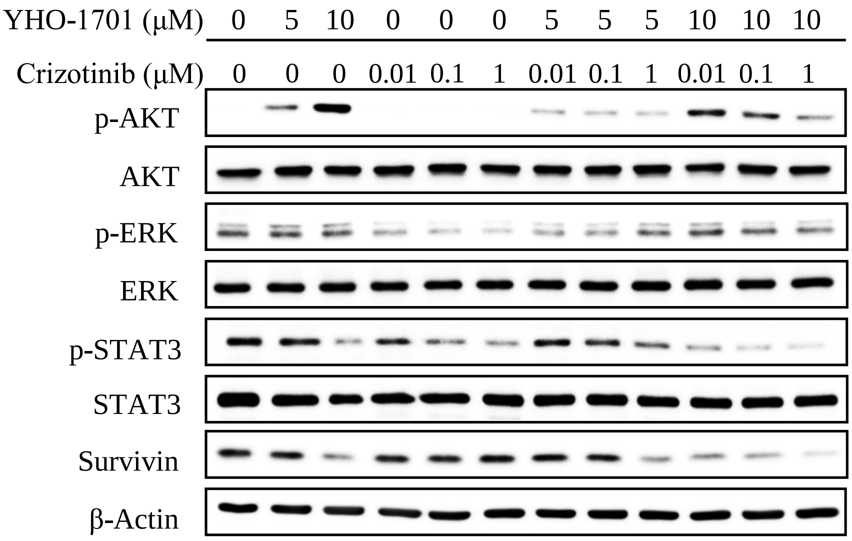

Supplementary Figure S2. Analysis of change in crucial molecules in each pathway when YHO-1701 was treated with crizotinib in H2228 cells.

Phosphorylation and activation of key molecules in the ALK and STAT3 signaling pathways were examined with western blotting. H2228 cells were left untreated or treated with YHO-1701 and/or crizotinib at the indicated doses for 24 h. The blots were probed with the respective primary antibodies. Cropped images are shown and the full-length blots are presented in Supplementary Fig. S3.

Supplementary Figure S3. Full-length images of blots presented in the main figures.

The full blot of p-BCR-ABL in Figure 3B

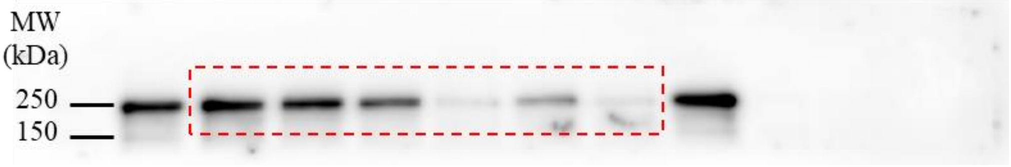

The full blot of BCR-ABL in Figure 3B

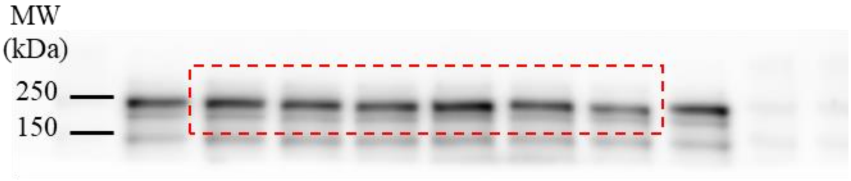

The full blot of p-STAT5 in Figure 3B

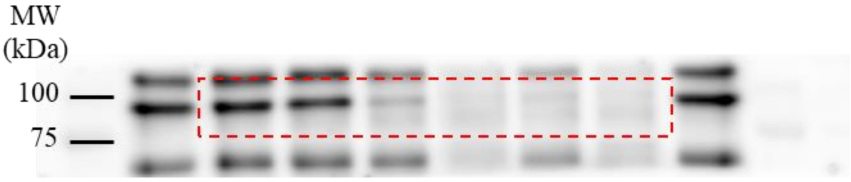

The full blot of STAT5 in Figure 3B

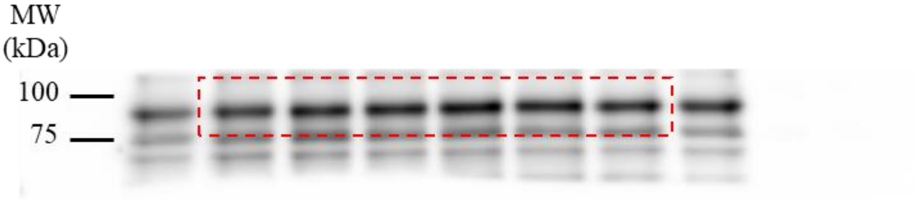

The full blot of p-STAT3 in Figure 3B

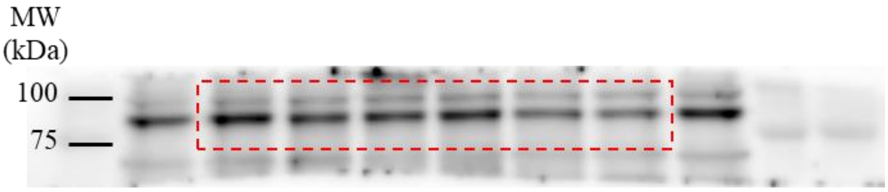

The full blot of STAT3 in Figure 3B [high contrast (upper); low contrast (lower)]

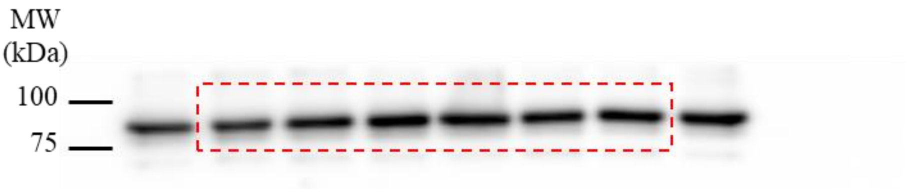

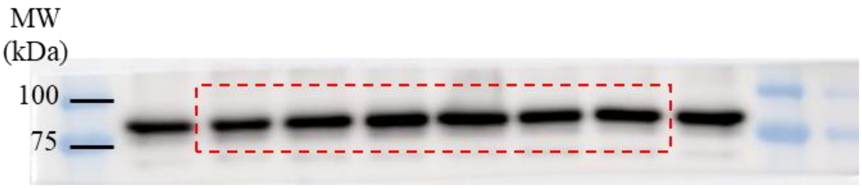

The full blot of survivin in Figure 3B

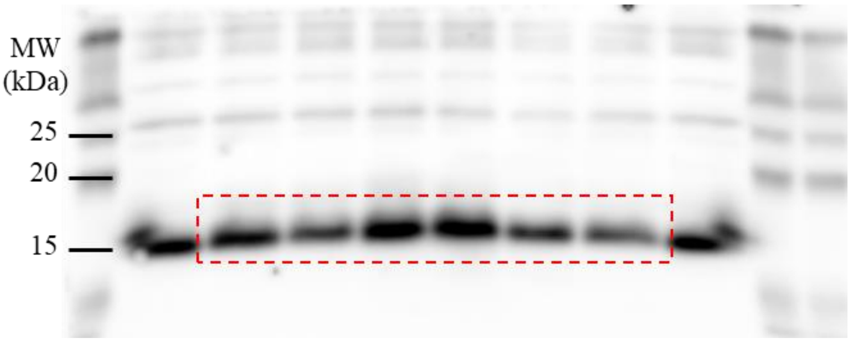

The full blot of β-Actin in Figure 3B [high contrast (upper); low contrast (lower)]

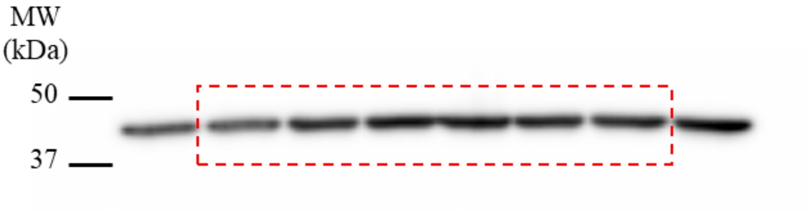

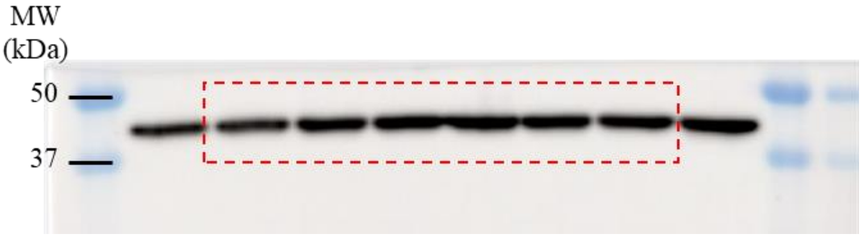

The full blot of p-EGFR in Figure 4B

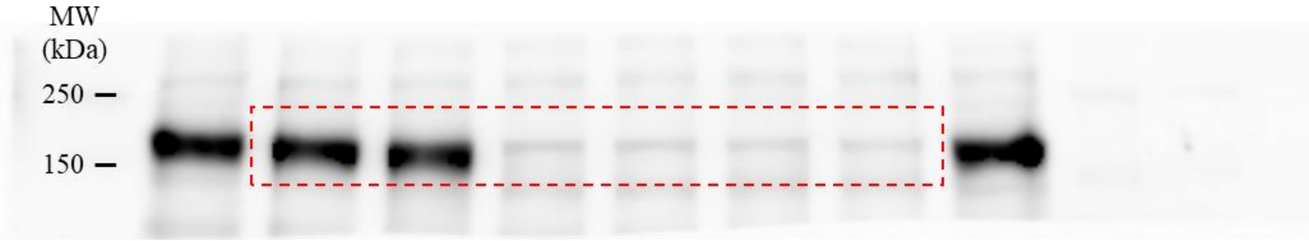

The full blot of EGFR in Figure 4B

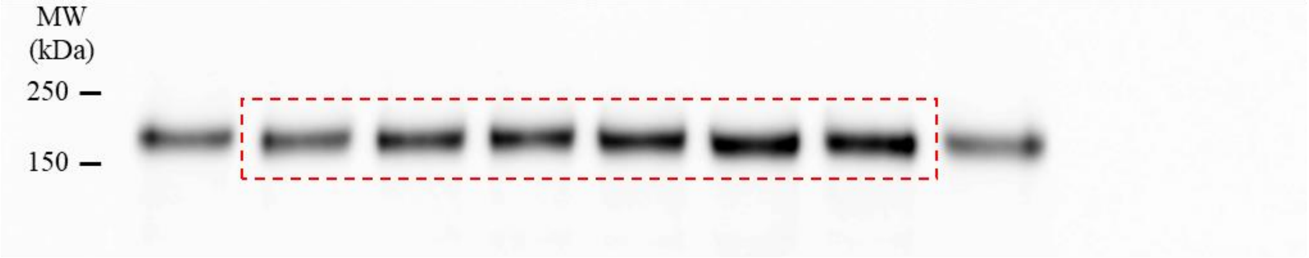

The full blot of p-AKT in Figure 4B

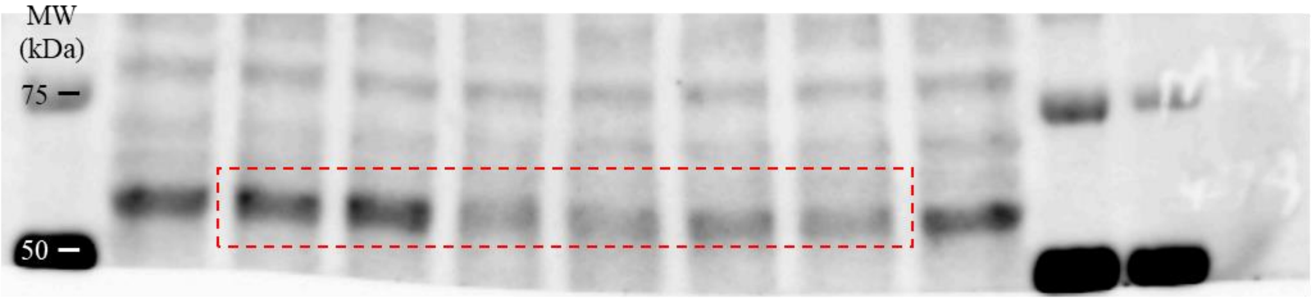

The full blot of AKT in Figure 4B

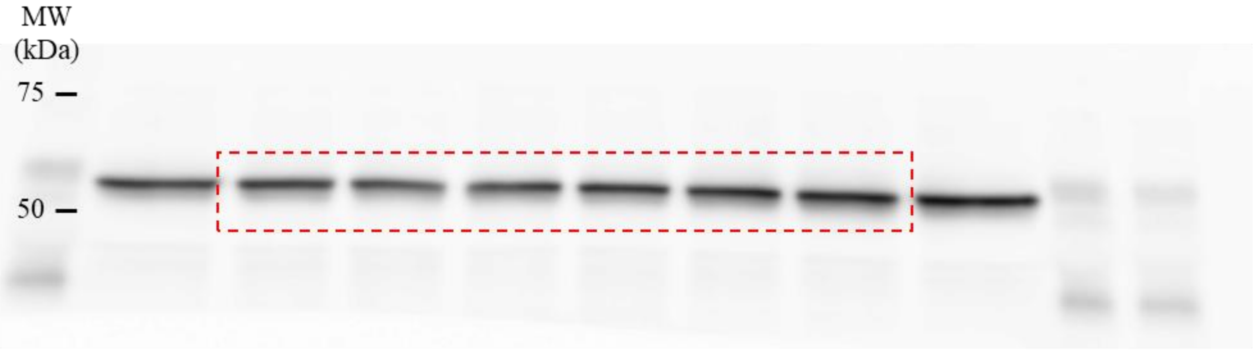

The full blot of p-ERK in Figure 4B

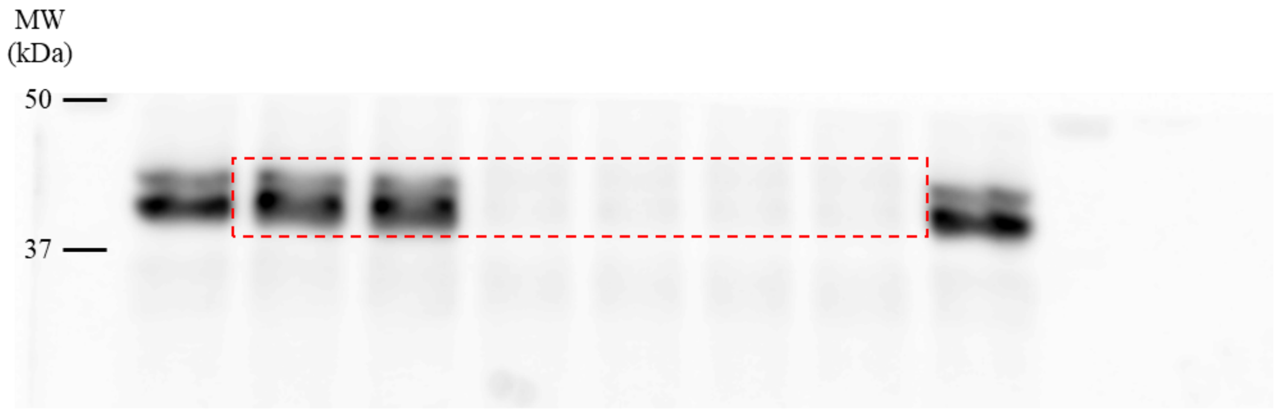

The full blot of ERK in Figure 4B

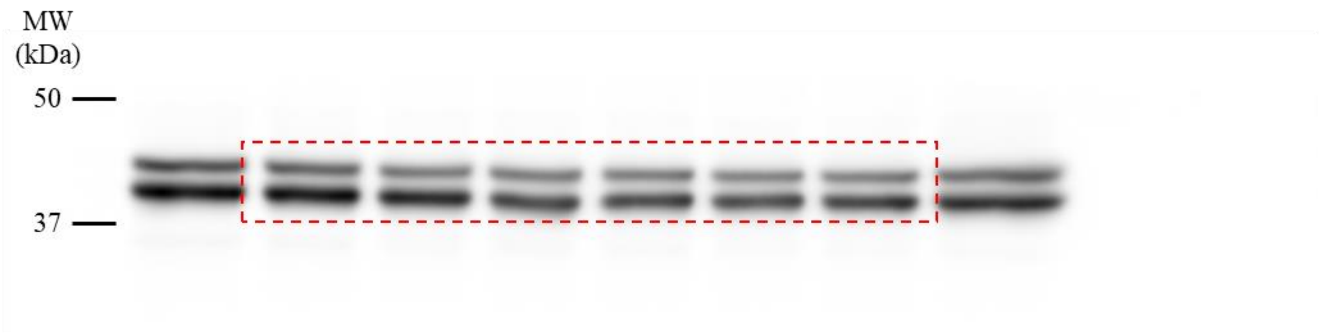

The full blot of p-STAT3 in Figure 4B

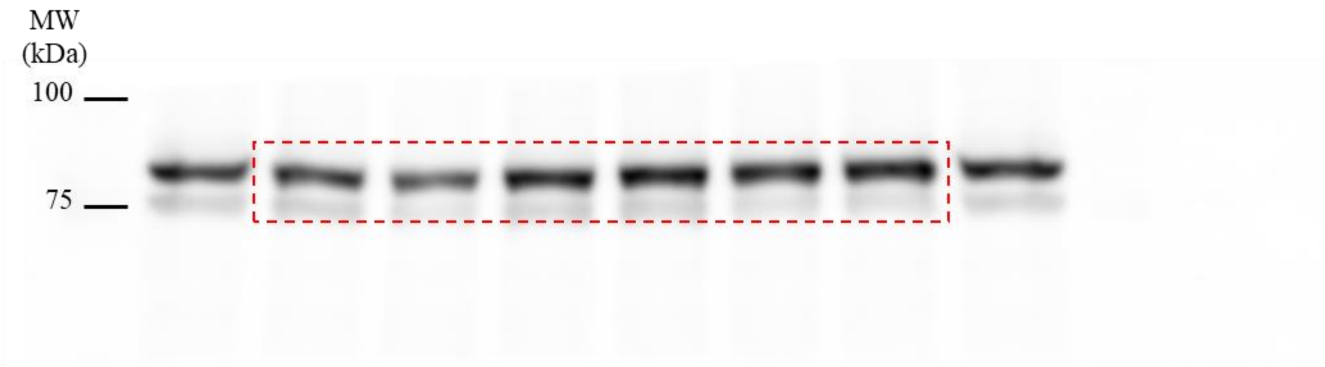

The full blot of STAT3 in Figure 4B

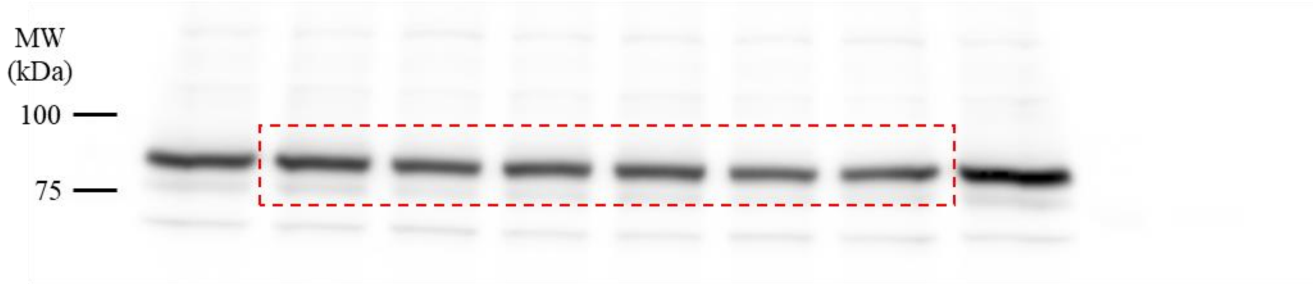

The full blot of survivin in Figure 4B

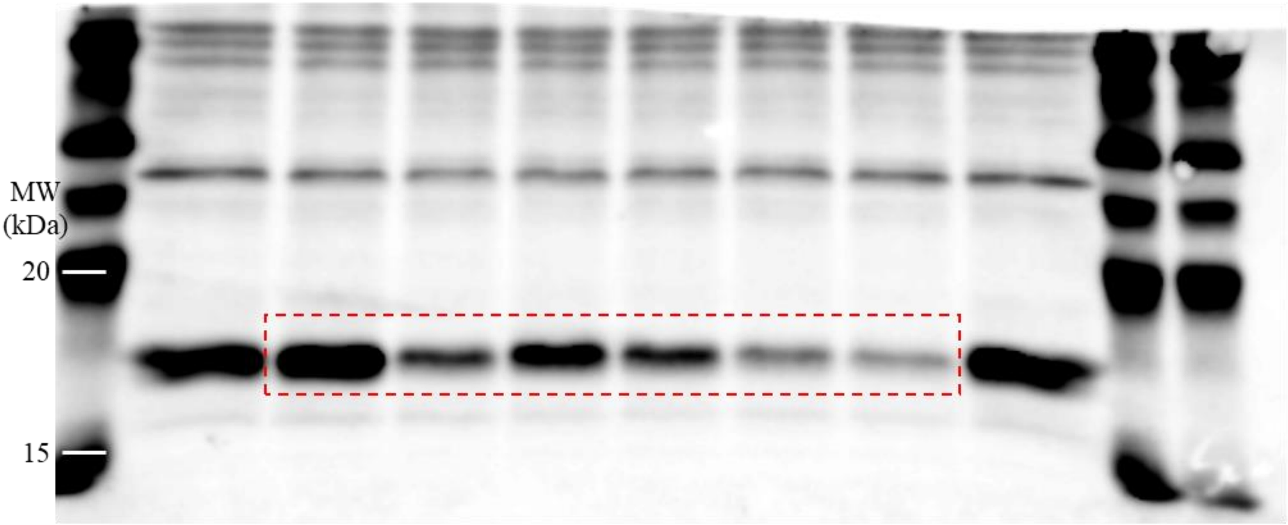

The full blot of β-Actin in Figure 4B

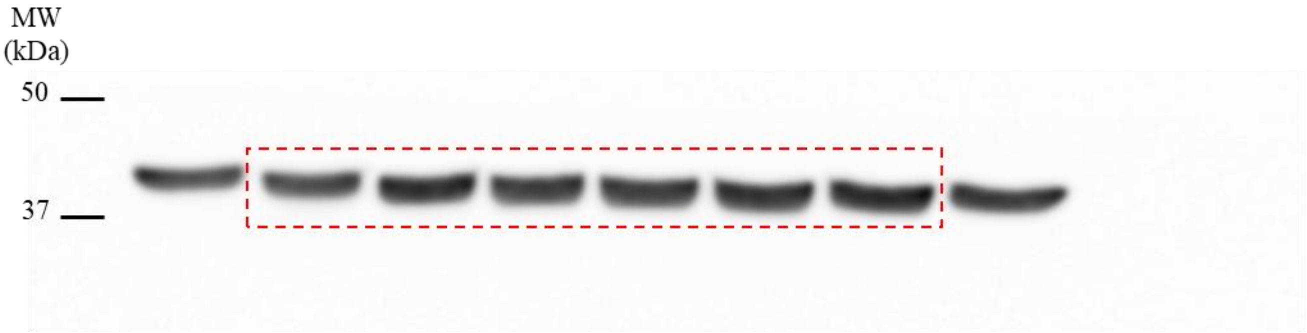

The full blot of p-ALK in Figure 5B

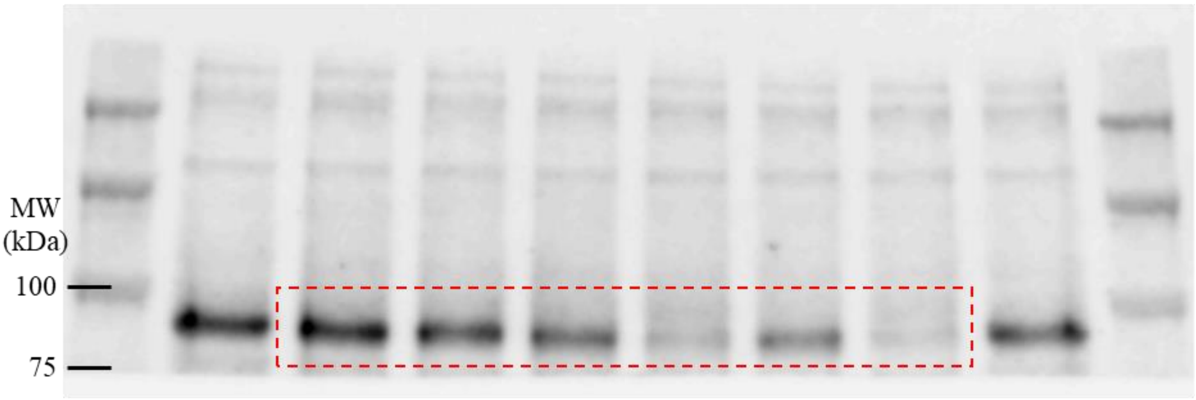

The full blot of ALK in Figure 5B

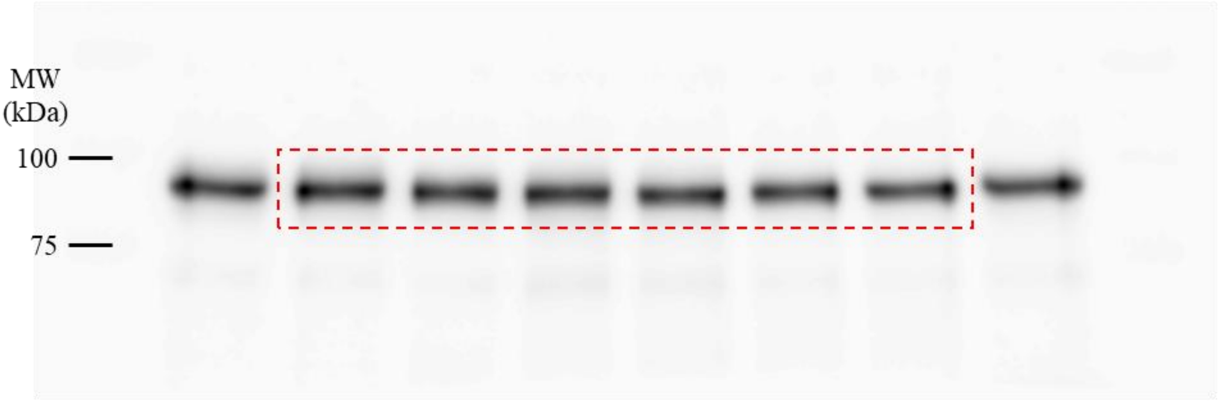

The full blot of p-AKT in Figure 5B

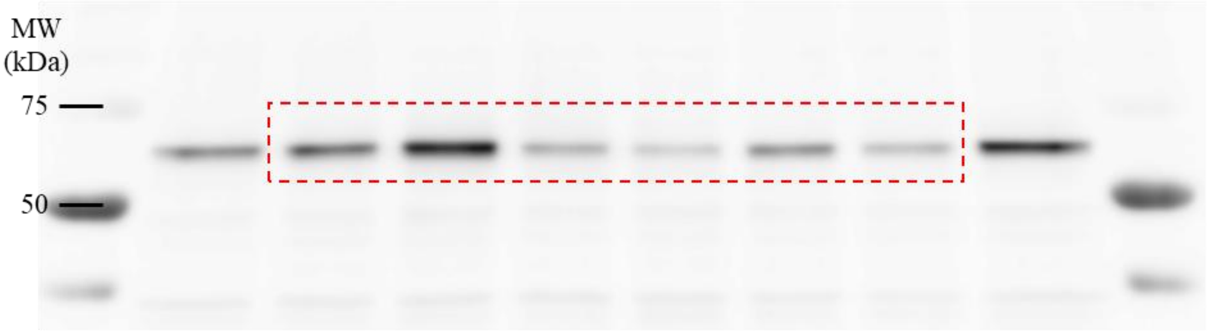

The full blot of AKT in Figure 5B

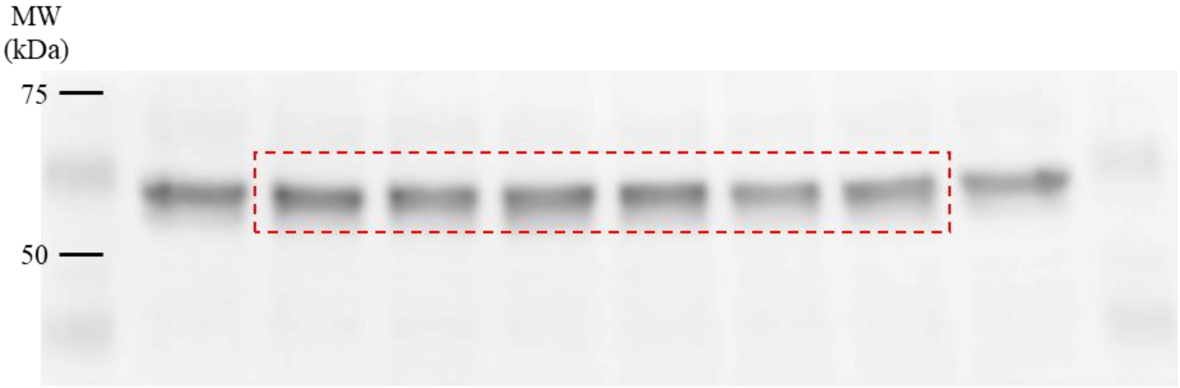

The full blot of p-ERK in Figure 5B

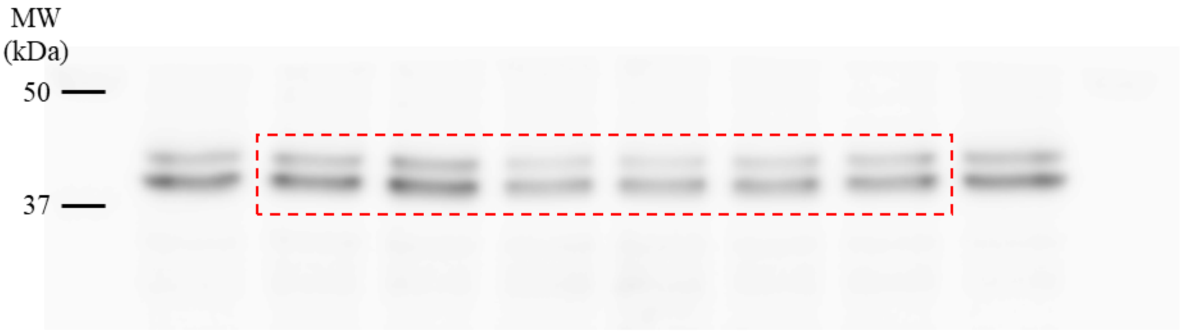

The full blot of ERK in Figure 5B

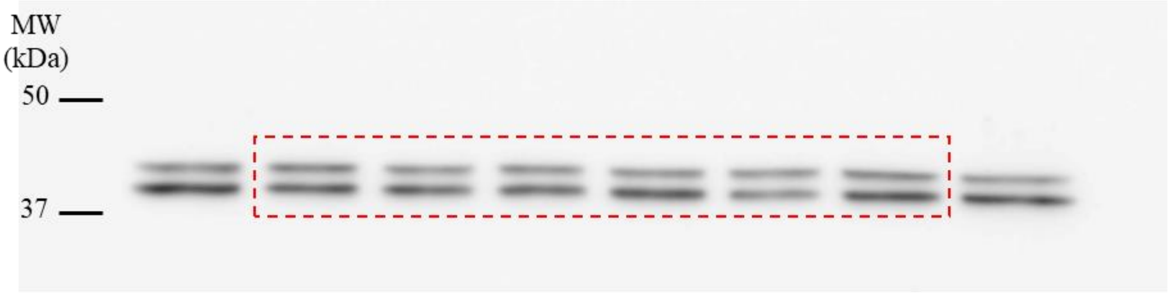

The full blot of p-STAT3 in Figure 5B

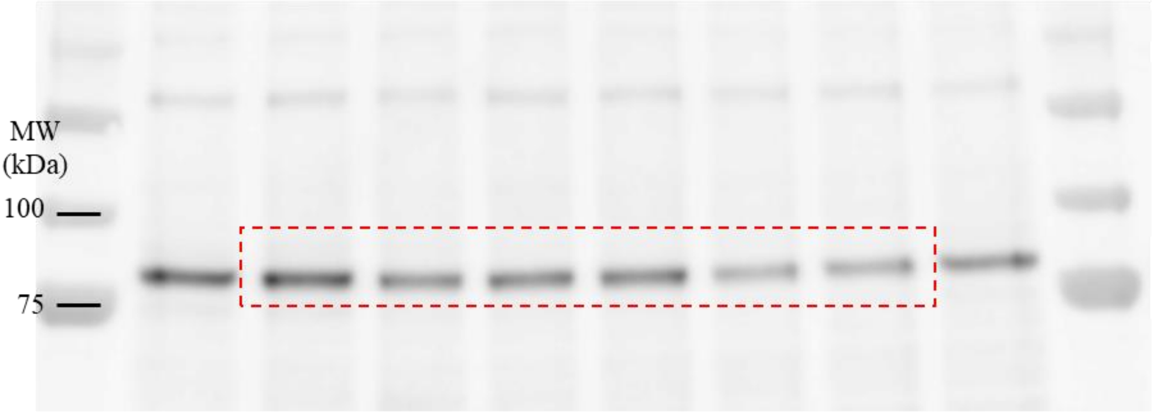

The full blot of STAT3 in Figure 5B

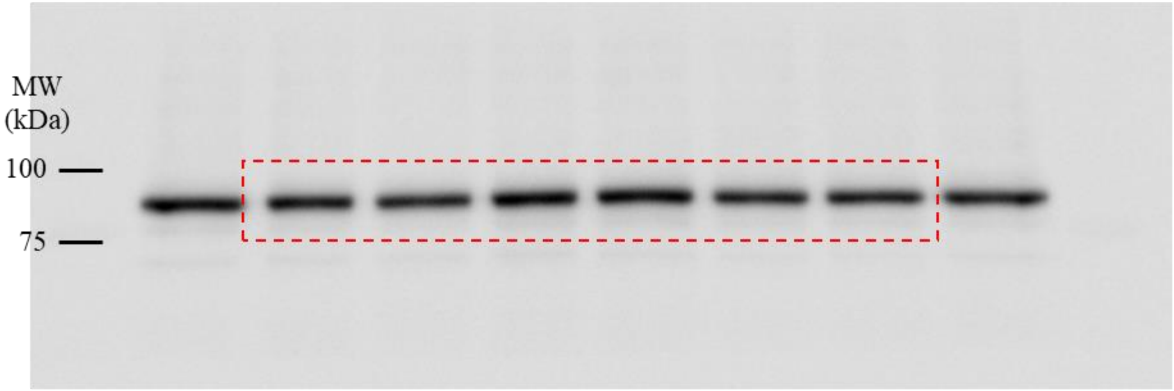

The full blot of survivin in Figure 5B

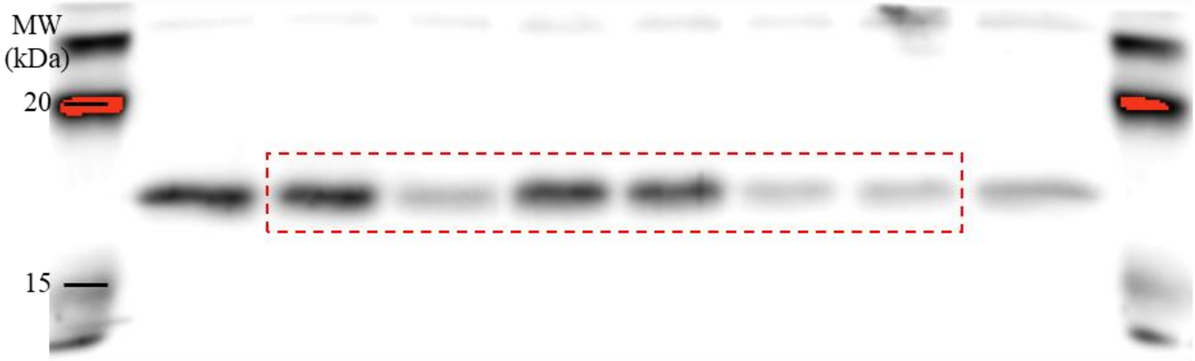

The full blot of β-Actin in Figure 5B

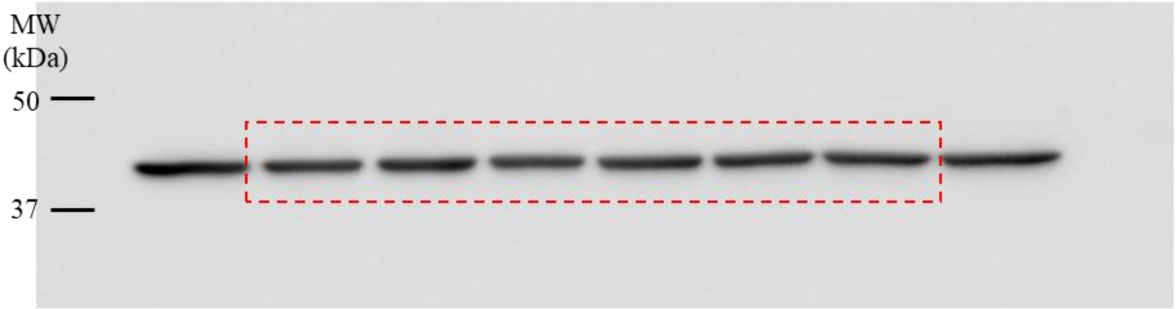

The full blot of p-AKT in Supplementary Figure S2 [high contrast (upper); low contrast (lower)]

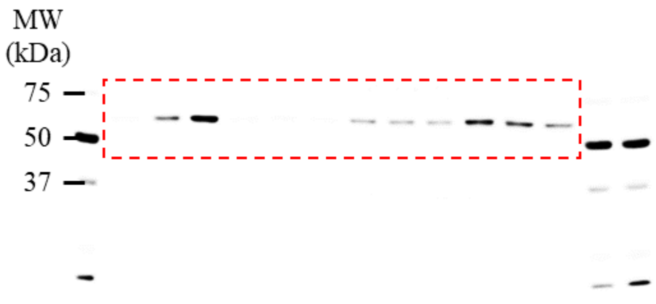

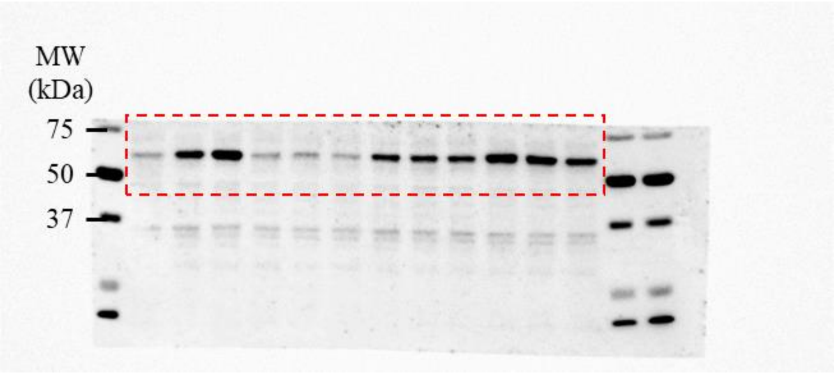

The full blot of AKT in Supplementary Figure S2 [high contrast (upper); low contrast (lower)]

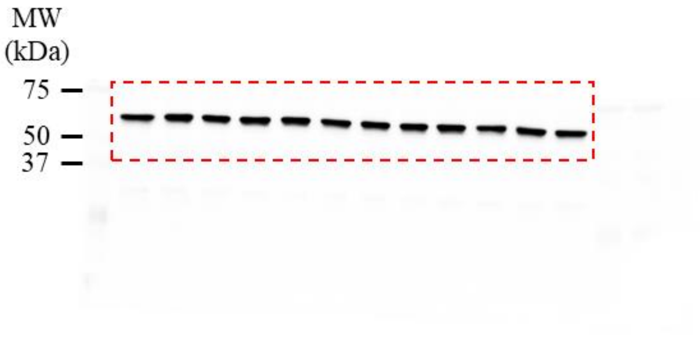

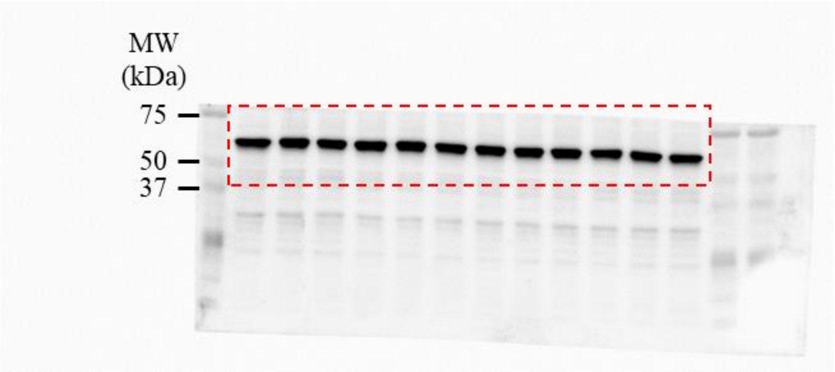

The full blot of p-ERK in Supplementary Figure S2 [high contrast (upper); low contrast (lower)]

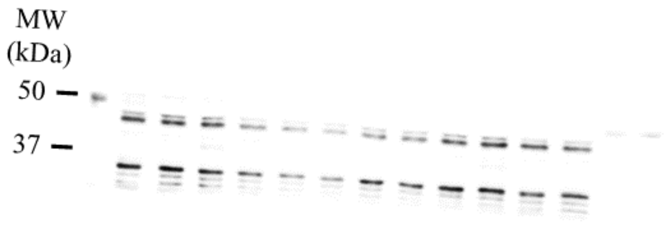

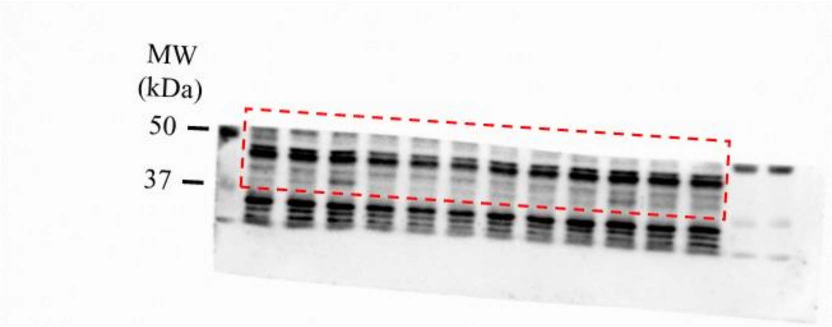

The full blot of ERK in Supplementary Figure S2 [high contrast (upper); low contrast (lower)]

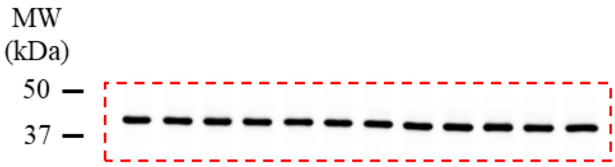

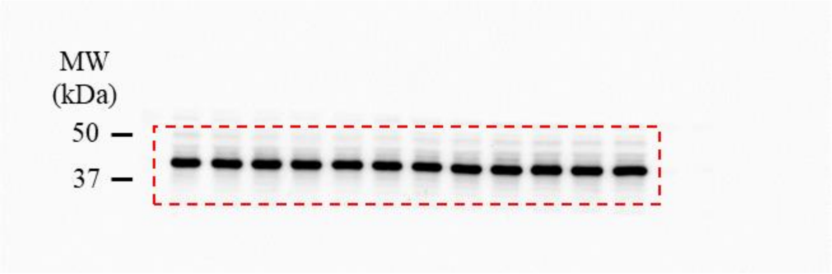

The full blot of p-STAT3 in Supplementary Figure S2 [high contrast (upper); low contrast (lower)]

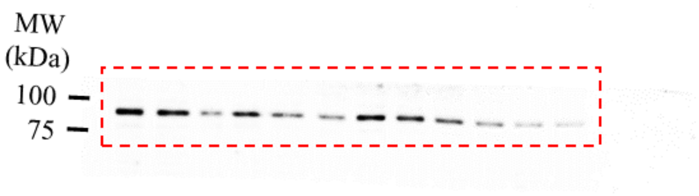

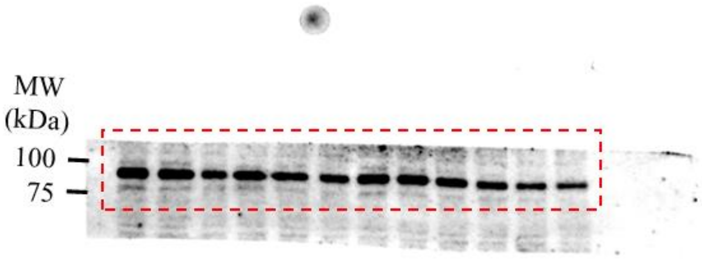

The full blot of STAT3 in Supplementary Figure S2 [high contrast (upper); low contrast (lower)]

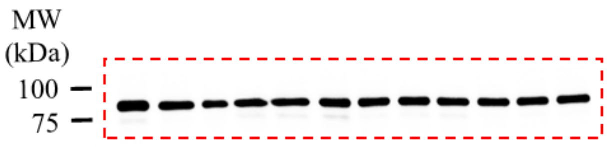

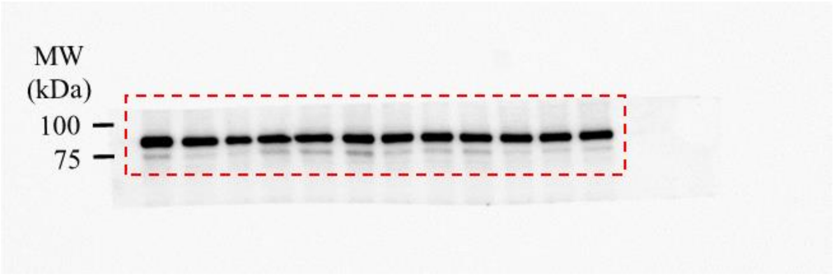

The full blot of survivin in Supplementary Figure S2 [high contrast (upper); low contrast (lower)]

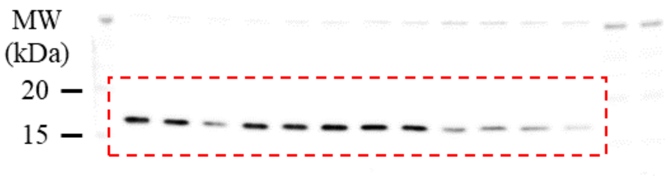

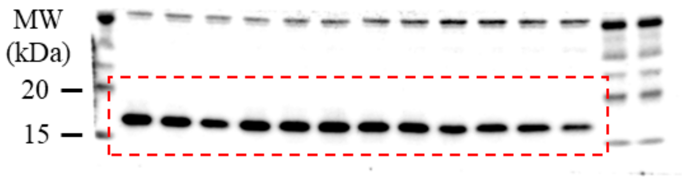

The full blot of β-Actin in Supplementary Figure S2 [high contrast (upper); low contrast (lower)]

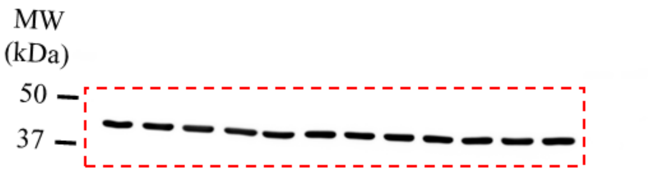

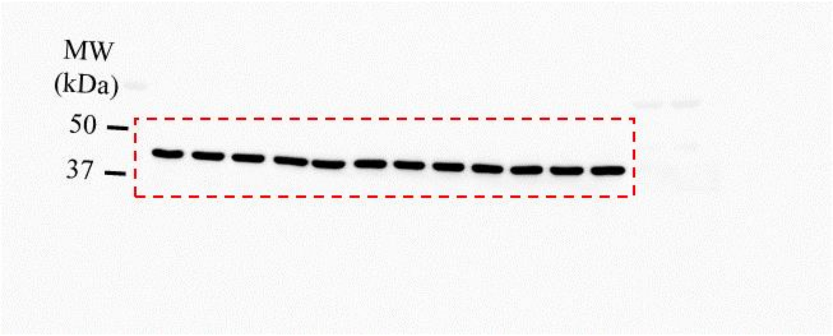

Supplement: Supplementary file 1 — Supplementary Information. [file 41598_2021_86021_MOESM1_ESM.pdf]
